# Neutrophil extracellular traps in bacterial infections and evasion strategies

**DOI:** 10.3389/fimmu.2024.1357967

**Published:** 2024-02-16

**Authors:** Ahmed Adel Baz, Huafang Hao, Shimei Lan, Zhangcheng Li, Shuang Liu, Shengli Chen, Yuefeng Chu

**Affiliations:** ^1^ State Key Laboratory for Animal Disease Control and Prevention, College of Veterinary Medicine, Lanzhou University, Lanzhou Veterinary Research Institute, Chinese Academy of Agricultural Sciences, Lanzhou, China; ^2^ Gansu Province Research Center for Basic Disciplines of Pathogen Biology, Lanzhou, China; ^3^ Key Laboratory of Ruminant Disease Prevention and Control (West), Ministry of Agricultural and Rural Affairs, Lanzhou, China; ^4^ Key Laboratory of Veterinary Etiological Biology, Ministry of Agricultural and Rural Affairs, Lanzhou, China; ^5^ Botany and Microbiology Department, Faculty of Science, Al-Azhar University, Assiut, Egypt

**Keywords:** neutrophil, neutrophil extracellular traps, bacterial infection, mycoplasma, NETs evasion, DNase

## Abstract

Neutrophils are innate immune cells that have a vital role in host defense systems. Neutrophil extracellular traps (NETs) are one of neutrophils’ defense mechanisms against pathogens. NETs comprise an ejected lattice of chromatin associated with histones, granular proteins, and cytosolic proteins. They are thought to be an efficient strategy to capture and/or kill bacteria and received intensive research interest in the recent years. However, soon after NETs were identified, it was observed that certain bacteria were able to evade NET entrapment through many different mechanisms. Here, we outline the recent progress of NETs in bacterial infections and the strategies employed by bacteria to evade or withstand NETs. Identifying the molecules and mechanisms that modulate NET release will improve our understanding of the functions of NETs in infections and provide new avenues for the prevention and treatment of bacterial diseases.

## 1 Introduction

Neutrophils are the most prevalent type of leukocytes circulating in the blood and are crucial for maintaining health. They differentiate from hematopoietic stem cells and are discharged from the bone marrow when terminally mature. They are the first cells to leave circulation and migrate to the infection site during an immunological challenge. There, they eliminate bacteria, communicate damage status to other immune cells, and are involved in the healing process (1). Much is known about the antimicrobial response of neutrophils. They are highly skilled in phagocytosis, which is their first vital mechanism for the eradication of disease-causing pathogens and elimination of dead cells and tissue debris (2, 3). The second mechanism for eliminating pathogens is degranulation, which involves regulation of the immune system response via the release of various granules (4–6).

Approximately 20 years ago, a new mechanism by which neutrophils fight infections was termed neutrophil extracellular traps (NETs) (7). This involves the loosening of chromatin in the neutrophil nucleus and forming complexes with granular and cytoplasmic proteins, which are then released into the extracellular environment. There, NETs encapsulate and kill microorganisms, and block their dissemination (7, 8).

## 2 NETs

NETs are a special form of programmed cell death, which is distinct from other cell death forms (apoptosis, necroptosis, and pyroptosis). In general, the complex process of NET formation begins with the recognition of microorganism, triggering the activation of the NETs pathway. This, in turn, leads to the breakdown of the nuclear membrane and the release of decondensed nuclear DNA into the cytoplasm. As this DNA mingles with cellular components including the histones, granules and cytoplasmic proteins, the process culminates in the rupture of the plasma membrane and the ultimate release of the NET structure into the extracellular space. Simultaneously, these traps are capable of degrading virulent bacterial factors (9–11).

### 2.1 NETs stimulation and signaling receptors

NET release is triggered by various stimuli. A range of bacterial species induce NET release. For Gram-positive bacteria, such as *Staphylococcus aureus* (7, 12, 13), *Streptococcus pyogenes* (14, 15), *Streptococcus pneumoniae* (16), *Streptococcus agalactiae* (17), *Streptococcus sanguinis* (18), *Streptococcus suis* (19). For Gram-negative bacteria, such as *Escherichia coli* (20–22), *Salmonella typhimurium* (7), *Shigella flexneri* (7), *Haemophilus influenzae* (23), *Pseudomonas aeruginosa* (24, 25), *Yersinia pseudotuberculosis* (26), *Photorhabdus luminescens* (27). In addition, other bacterial species, including *Mycobacterium tuberculosis* (28, 29)*, Mycobacterium canettii* (28), *Mycobacterium avium* (30), and *Mycoplasma bovis* (31). Furthermore, bacterial components like lipopolysaccharide (LPS) (7), flagella (25), bacterial toxin nigericin (17), and calcium ionophore A2318 (17) are also involved in NETs stimulation. Additionally, diverse inflammatory mediators including interferon (IFN)-α (32), IFN-γ (32), interleukin (IL)-8 (7), IL-1β (33, 34), IL-2 (35), IL-6 (36), IL-10 (36), tumor necrosis factor (TNF)-α (33, 37), granulocyte-macrophage colony-stimulating factor (GM-CSF) (38), transforming growth factor (TGF)-β (34), and complement factor 5a (C5a) (32, 38) have been shown to stimulate NET release.

Moreover, Neutrophil receptors play a crucial role in activation of neutrophil and formation of NETs. The specific engagement of cell receptors by extracellular signaling molecules activates diverse intracellular signaling cascades and regulate NETs functions. In response to different bacterial triggers, NETs can release by the activation of different receptors including toll-like receptors (TLRs) (39–44), nucleotide-binding oligomerization domain-like receptors (NLRs) (45), C-type lectin receptors (CLRs) (46), complement receptors (CRs) (47), Fc receptors (47–50) and other neutrophil receptors like protease activated receptor-2 (PAR-2) (51).

### 2.2 NET components

The composition of NETs varies depending on the stimuli, and is primarily made up of DNA and histone proteins (H1, H2A, H2B, H3, and H4), which are originated from the nucleus followed by granular proteins that are normally stored in distinctive neutrophil granules in the cytoplasm of neutrophil such as neutrophil elastase (NE), myeloperoxidase (MPO), defensins, and cathepsin G from primary granules, lactoferrin, cathelicidins, and lysozyme C from secondary granules, and gelatinase from tertiary granules, as well as other proteins such as calprotectin and proteinase 3 are located in the cytoplasm of neutrophils (7, 52–54) (**Supplementary Table S1**).

### 2.3 Mechanisms of NETs formation

NETs were first reported in 2004 and were primarily described as a cell death process in activated neutrophil (7). When DNA leaks extracellularly, not all NET developments result in cell death, and not every neutrophil death necessarily results in NET formation. “NETosis” is often employed throughout research for referring to NET formation, which can be categorized to as “lytic” and “non-lytic” forms.

#### 2.3.1 Lytic mechanism

The “lytic” mechanism of NET formation is the interaction between stimuli and cell receptors, which triggers Raf-MEK-ERK signaling and activates the NADPH oxidase complex (55). This complex produces peroxide ions in neutrophils, increasing levels of cytosolic reactive oxygen species (ROS). MPO detects this rise and is often paired with other proteases to form the azurosome. MPO trigger the activation and translocation of NE from azurophilic granules to the nucleus, where elastase proteolytically processes histones to disrupt chromatin packaging (56, 57). MPO then binds to chromatin and synergizes with NE to decondense chromatin independently of its enzymatic activity (57). The nucleus expands along with its chromatin, and cell lysis occurs because of gasdermin D, which can be activated by serine proteases in neutrophil inducing pore formation in the cell membrane (58). NETs are extruded into the extracellular space after membrane rupture and cell death within 3–8 h of neutrophil activation (9, 59).

#### 2.3.2 Non-lytic mechanism

The “non-lytic” or vital mechanism, involves increasing the concentration of cytosolic calcium through calcium ionophores, which activate the protein arginine deiminase 4 (PAD4) that leads to citrullates arginine histone residues, and reduces their positive charge. Histones then begin to lose their electrostatic attraction to DNA, and the chromatin expands along with the nucleus (37, 60). This oxidase-independent mechanism occurs within minutes of gram-positive skin infections; the cells release NETs during crawling without lysis, which prevents systemic bacterial dissemination (13, 61). However, vital NET formation might be more closely associated with infection than previously thought because soon after releasing NETs, neutrophils are still viable and can carry out additional host response processes, such as phagocytosis, chemotaxis, and microbial killing (62). In addition to non-lytic NETs with nuclear DNA release, NETs composed of mitochondrial DNA, NE, and are first described in GM-CSF-primed neutrophils stimulated *in vitro* with LPS or C5a (38, 63) since this process would not require neutrophil lysis, which would enable phagocytosis to continue (10).

#### 2.3.3 Other forms of NETs

However, divergent views have persisted in this field. NETs can form in the absence of PAD4 and citrullinated histone 3 (64, 65). It can also occur during leukotoxic hypercitrullination (LTH), defective mitophagy, and organ injury (66). Other types of NETs have also been described, including cloudy NETs, spiky NETs, aggregated NETs, and bicarbonate-induced aggregated NETs (67). Therefore, we cautiously take opinions about these studies into consideration

## 3 Effects of NETs in bacterial infections

### 3.1 Morphological effects

NETs have various effects on the morphology of bacteria, including physically trapping and immobilizing them, leading to changes in their morphology as they become entangled in the web-like structure of the NETs (7). Additionally, they can release DNA, enzymes, and antimicrobial proteins that can damage the cell walls of bacteria and/or membranes, leading to changes in their morphology as they become structurally compromised (68–70). Furthermore, NETs are involved in the disruption of bacterial biofilms, which can also lead to changes in the morphology of the bacteria (71).

### 3.2 Functional effects

Along with the impact of NETs on the shape of bacteria, they can kill and limit the growth of bacteria and prevent their spread in the environment. They possess antimicrobial properties with components such as DNA, histones, granules, and cytoplasmic proteins that have bactericidal effects (7, 68, 72–75). Interestingly, NETs play an important role in the defense against bacteria, even in the absence of microbicidal activity. These infections are ensnared but not eliminated by NETs (28, 76–79), indicating that NET-mediated microbial trapping alone plays a substantial role in immune defense.

As mentioned above, the formation of NETs and their effect on bacterial infection are mostly reported *in vitro* conditions. However, there is limited research describing the stimulation of NETs in response to bacterial infection *in vivo*. It is difficult to verify NETs *in vivo* because it calls for specialized technical knowledge. Besides, it is hard to assess the kind of stimulus, its dosage, and its exposure duration *in vivo*. Researchers may use genetically modified animal models to study the role of specific proteins or signaling pathways in the regulation of NET formation during bacterial infection (80).

Furthermore, the composition of NETs determines their efficiency. Mice exposed to low concentrations of cathepsin G are more susceptible to infection with the Gram-positive bacterium *S. aureus* (81), whereas those exposed to low concentrations of NE are more susceptible to Gram-negative bacteria such as *E. coli*, *K. pneumoniae*, and other *Enterobacteriaceae* (70, 81). Additionally, the antimicrobial response of NETs is influenced by the environment in which they are developed; NETs under static conditions show limited bacterial killing, whereas those under dynamic conditions show enhanced bacterial trapping and reduced killing (78).

Although NETs protect the host against microbes, there are diseases and conditions that can interfere with the release of NETs. Some examples of diseases that can affect NET release or neutrophil function are autoimmune diseases, chronic inflammatory conditions and sepsis, which can alter the function of neutrophils and their ability to release NETs. In these conditions, the dysregulation of neutrophil function and NET release can contribute to the pathology of the disease and affect the body’s ability to fight infections and maintain immune homeostasis (82, 83).

## 4 Modulation of NET release in bacteria

As mentioned before, the release of NETs is essential for defense against pathogens; the evasion of NETs appears to be a widespread strategy to allow pathogen proliferation and dissemination and is currently a topic of intense research interest. Here, we review the current knowledge of evasion strategies used by bacteria to dysregulate NET formation and functions.

### 4.1 NET formation inhibition

Specific molecules and pathways that inhibit NET release. Thus, NET inhibition mechanisms in bacterial infections are summarized in this section of the review (**Table 1**).

**Table 1:** Factors associated with the inhibition of NET release in bacterial infections.

Evasion strategy	Molecule (s)	Microorganism	Modulatory effect on NETs formation	References
Inhibition	Flagella	*P. aeruginosa*	Flagellum-deficient bacteria are severely impaired in triggering NET formation	(25)
	LasR		*P. aeruginosa* lacking LasR have a restricted ability to cause the release of neutrophil DNA.	(84)
	Sialoglycoproteins		Display binding to Siglec-9, reduce ROS level and elastase release, and lower the development of NETs	(85)
	SpyCEP	GAS	Cleaves IL-8, and reduces neutrophil production of extracellular traps	(86)
	SLO		Blocking IL-8 secretion and responsiveness & Dissolve cell membranes and fibrous extracellular DNA strands	(87)
	HMW-HA		Engage hSiglec-9, block oxidative burst and NET formation	(88)
	?	*M. catarrhalis*	Suppresses the ROS production, and thus inhibiting the production of NETs	(89)
	TcpC	*E. coli*	Promoting the degradation of PAD4, and reduce NET formation	(90)
	Sialylated polysaccharide	GBS	Binding to Siglec-9, suppressing ROS and reduce formation of NETs	(91)
	CPS		Binding to Siglec-5, inhibits oxidative burst, and impair NETs formation	(92)
	ACT	*B. pertussis*	Inhibit the oxidative burst by generating cAMP and consequently inhibit formation of NETs	(93)
	CyaA	*B. parapertussis*	Mediated inhibition of ROS and reduces NET activation	(94)
	LPS	*K. pneumoniae*	Involve in ROS inhibition and diminish the formation of NETs	(95)
	T3SS and CPS-I	*B. pseudomallei*	Inhibition of NADPH oxidase pathway and reduce NET release	(96)
	?	*A. baumanii*	Suppression of the surface expression of CD11a in neutrophils	(97)
	WiP1	*S. aureus*	Display suppression of NET release	(98)

*P. aeruginosa*, *Pseudomonas aeruginosa*; *M. catarrhalis*, *Moraxella catarrhalis*; *E. coli*, *Escherichia coli*; *B. pertussis*, *Bordetella pertussis*; *B. parapertussis*, *Bordetella parapertussis*; *K. pneumoniae*, *Klebsiella pneumoniae*; *B. pseudomallei*, *Burkholderia pseudomallei*; *A. baumanii*, *Acinetobacter baumannii*; *S. aureus*, *Staphylococcus aureus*; ROS, reactive oxygen species; IL-8, interleukin-8; SLO, streptolysin O; GAS, group A *Streptococcus*; HMW-HA, high molecular weight-hyaluronic acid; PAD4, protein arginine deiminase 4; GBS, group B *Streptococcus*; HMW-HA, high molecular weight-hyaluronic acid; PAD4, protein arginine deiminase 4; GBS, group B Streptococcus; Siglec-9, Sialic acid-binding Ig-like lectin-9; Siglec-5, Sialic acid-binding Ig-like lectin-5; CPS, capsular polysaccharide; ACT, adenylate cyclase toxin; cAMP, cyclic adenosine monophosphate; CyaA, adenylate cyclase; LPS, lipopolysaccharide; T3SS, type 3 secretion system; CPS-I, capsular polysaccharide-I; NADPH, nicotinamide adenine dinucleotide phosphate; WiP1, wild-type p53-induced phosphatase 1; ?, unknown.

#### 4.1.1 Downregulation of NET-stimulating phenotypes

Suppression of phenotypes that trigger NET release is a way to prevent their formation. Flagellum is the main bacterial component required to trigger maximal NET release, and flagellum-deficient bacteria remain seriously impaired in triggering NET formation (25). In addition, LasR-deficient *P. aeruginosa* strains harbor a limited capability to trigger neutrophil DNA release due to reduce the expression of elastase LasB and proteases LasA. The neutrophil NADPH oxidase pathway is required to decrease NET release caused by LasR-deficient strains, but it is not dependent on downstream quorum sensing pathways, LPS synthesis, or bacterial motility (84).

#### 4.1.2 Inhibition of NET-triggered molecules

IL-8 is a prominent neutrophil chemoattractant and activator that induce NET formation (7). However, group A *Streptococcus* (GAS) protease SpyCEP (also called ScpC) cleaves IL-8 and reduces neutrophil production of extracellular traps (86). In addition, streptolysin O toxin (SLO) prevents the release of IL-8 and elastase from neutrophils, blocks NET formation, and inhibits NETs by dissolving cell membranes and fibrous extracellular DNA strands (87).

#### 4.1.3 Suppression of NET-mediated receptors on neutrophil

Neutrophil surface receptors have been linked to suppression of NET formation. GAS expresses a high-molecular-weight hyaluronic acid capsule (HMW-HA). hSiglec-9 specifically binds to HMW-HA through a region of its terminal Ig-like V-set domain distinct from the Sia-binding site. HMW-HA recognition by hSiglec-9 blocks the oxidative burst and limits NET formation, thereby promoting bacterial survival (88). Moreover, the sialylated capsular polysaccharide of Group B *Streptococcus* (GBS) interacts with neutrophil Siglec-9, dampening neutrophil responses in a Sia-and Siglec-9-dependent manner, causing a reduction in the neutrophil oxidative burst, diminished formation of NETs, and increased bacterial survival (91). In addition, β protein from GBS inhibits human leukocyte phagocytosis, oxidative burst, and extracellular trap formation by binding to hSiglec-5 in a Sia-independent manner (92). However, engagement of Siglec−14 by β−protein antagonizes the repressive effects of Siglec−5 by activating mitogen-activated protein kinase (MAPK) signaling (99). *Moraxella catarrhalis* limits ROS production by possibly binding to immunosuppressive Siglecs receptors (Siglec-5 and Siglec-9) and consequently suppressing NET generation (89). Furthermore, soluble Siglec-9 exhibited strong binding with α2-3-linked sialoglycoproteins adsorbed by *P. aeruginosa*. The interaction between *P. aeruginosa* (+Sias) and siglec-9 on neutrophils decreases the amount of ROS and the release of elastase, which in turn decreases the formation of NETs (85). Moreover, *Acinetobacter baumannii* inhibits the formation of NETs by suppressing the surface expression of CD11a in neutrophils, thereby escaping host immune responses and contributing to the development of *A. baumannii* infections (97).

#### 4.1.4 Interfering of NADPH signaling and ROS formation

NET formation is related to the production of ROS, both cytosolic and mitochondrial (55, 100). The adenylate cyclase toxin (ACT) of *Bordetella pertussis* inhibits formation of NETs by generating cyclic adenosine monophosphate (cAMP), consequently inhibiting oxidative burst (93). *Bordetella parapertussis* also expresses CyaA in a Bvg-regulated manner. This toxin is released into the extracellular space, which mediates the inhibition of ROS and reduces NET activation in human-derived neutrophils (94).

*K. pneumoniae* ST258 is a poor inducer of ROS generation, and consequently inhibits NET formation, suggesting that the polysaccharide part of LPS is responsible for this inhibition and results in increased bacterial survival (95). *Burkholderia pseudomallei* modifies the magnitude of NET formation via the action of its type 3 protein secretion system (T3SS), encoded by the *bsa* locus, and capsular polysaccharide I (CPS-I) encoded by the *wcb* operon, which attenuates NET release by inhibiting the NADPH oxidase pathway. *B. pseudomallei* mutants defective in the virulence-associated T3SS or CPS-I induced elevated levels of NETs. NET induction by these mutants is associated with increased bacterial killing (96).

#### 4.1.5 Others

Wild-type p53-induced phosphatase 1 (Wip1) suppresses NET release in *S. aureus* in mice model. Inhibition of Wip1 significantly suppresses the activity of *S. aureus* and accelerates abscess healing in *S. aureus*-induced abscess model mice by enhancing NET formation (98). Moreover, uropathogenic *E. coli* secretes a multifunctional virulence factor called TcpC, which primarily inhibits NETs by serving as an E3 ligase, promoting the degradation of PAD4. TcpC not only inhibits the citrullination of chromatin histones, but also affects the transcription of related genes in the nucleus and represents an additional NET evasion function of this bacterial derived virulence factor (90).

### 4.2 Degradation of NET components

**Figure 1:**
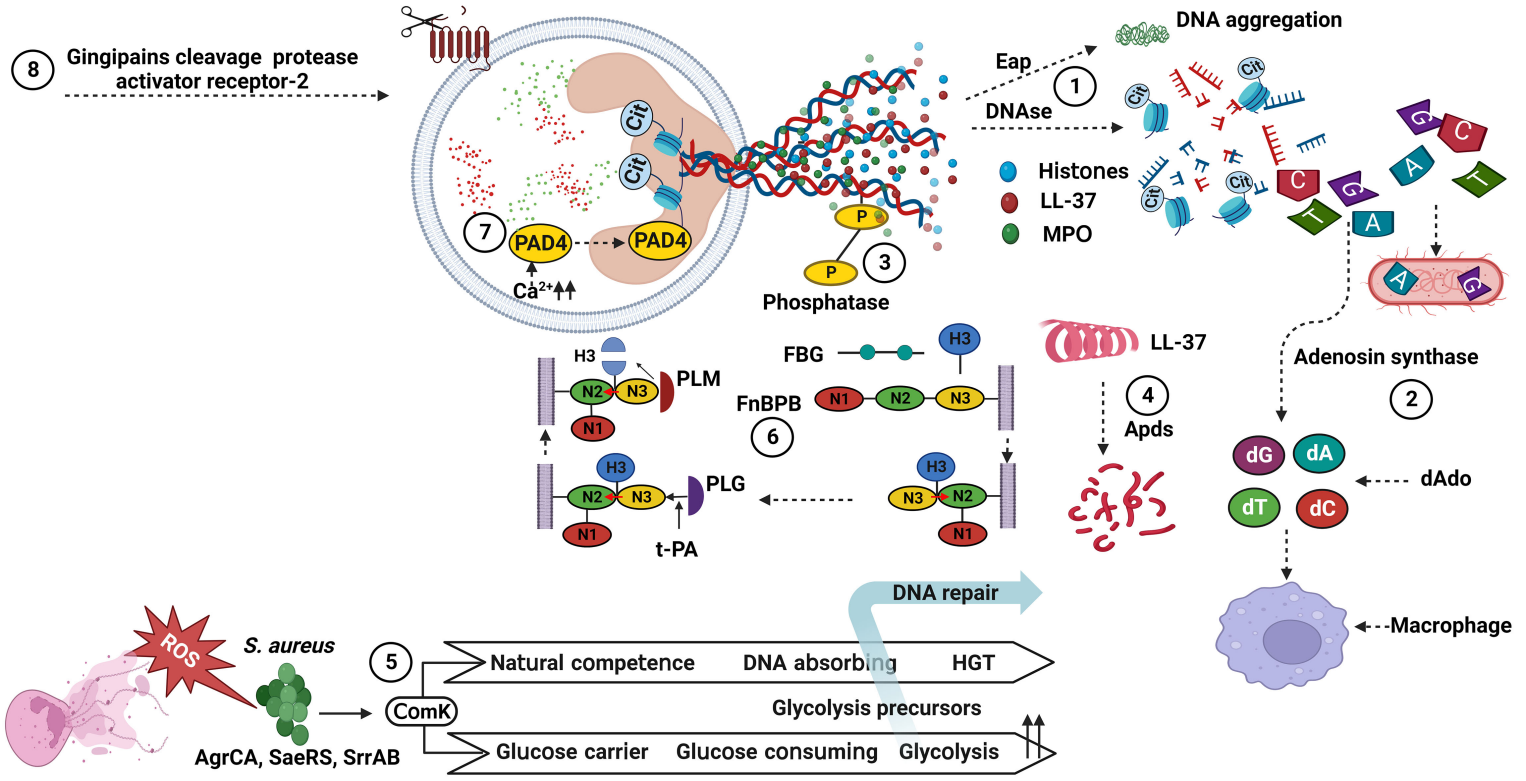
Bacterial factors associated with the degradation of NET components. (1) DNAses cleave NET-associated DNA, and Eap bind to the termini of linearized DNA and expresses an intrinsic DNA aggregation activity. (2) Adenosine synthase act together with the nuclease for generating deoxyadenosine fragments that are apoptotic for macrophages. (3) Phosphatase remove phosphate group from DNA, showing a cation chelating effect. (4) Apds cleave N-terminal amino acid from cathelicidin LL-37 and lose its helical structure. (5) ComK increase glucose and DNA uptake and downregulate ROS production. (6) FnBPB, in preference to FBG, H3 binds to the tunnel between the N2 and N3 of FnBPB, and then causes a conformational shift that allow the complex to stabilize by insertion of the terminal residues of the N3 extension (red arrow). Next, PLG binds to a N3 site, activated by t-PA to PLM, and cleaves FnBPB-bound H3. (7) PAD4 change histone arginines into histone citrullines, and losing its interactions with bacterial membranes. (8) Gingipains, cysteine protease, cleave protease-activated receptor-2 on the neutrophil surface. dAdo, deoxyadenosine; ROS, reactive oxygen species; HGT, horizontal gene transfer; PAD4, protein arginine deiminases 4; MPO, myeloperoxidase; FnBPB, fibronectin binding protein B; FBG, fibrinogen; H3, histones 3; PLG, plasminogen; t-PA, tissue plasminogen activator; PLM, plasmin; Eap, extracellular adhesion protein; Cit, citrullin. *S. aureus, Staphylococcus aureus*. Created with BioRender.com.

Studies investigating the production of NET-degrading molecules by bacteria have focused largely but not exclusively on the nucleases (**Table 2**; **Figure 1**).

**Table 2:** Lists the molecules for degradation of NET release in bacterial infections.

Evasion strategy	Molecule (s)	Microorganism	Modulatory effect on NETs formation	References
Degradation	Nuc	*S. aureus*	Escaping from NET-mediated killing and facilitates increased survival of *S. aureus*	(101, 102)
	Eap		Aggregate extracellular DNA and protecting the bacteria from NETs	(103, 104)
	Ads		Conversion of NETs to deoxyadenosine, which triggers-mediated death of immune cells	(105, 106)
	ArlRS-MgrA		Control NET-evasion mechanisms and regulate nuclease expression that involved in degradation of NETs	(107)
	ComK		Upregulate glucose and DNA-uptake and downregulate ROS production	(108)
	FnBPB		Bind to histone H3 and plasminogen, and cleave the bound histone	(109)
	EddB	*P. aeruginosa*	Degrade extracellular DNA to defend against NETs	(110)
	EddA		Removing the cation-chelating phosphates from the extracellular DNA phosphodiester backbone	(110)
	PAD4		Citrullination of extracellular histone H3.1	(111)
	Sda1	GAS	Enables bacteria to evade the host immune response by degrading the DNA backbone of NETs	(77, 112)
	SpnA			(113)
	endA	*S. pneumoniae*	Allows pneumococci to degrade the DNA scaffold of NETs	(76, 114)
	TatD			(115)
	SsnA	*S. suis*	They favor for the degradation of NETs	(116)
	endA			(117)
	ApdS		Cleave cathelicidin LL-37, impairs its ability to promote NETs formation and ROS production	(118)
	SWAN	*S. sanguinis*	Contributes to escape and degradation of NETs	(119)
	Thermonuclease	*N. gonorrhoeae*	Enables bacterium to escape from NET-mediated trapping and killing	(120)
	Nuc	*Y. enterocolitica*	Acts as Ca^2+^/Mg^2+^-dependent NET-degrading enzyme	(121)
	Dns and Xds	*V. cholera*	Degrade the DNA of NETs via the combined activity of the two extracellular nucleases	(122)
	Nuc	*Leptospira* spp.	Enable to degrade the NET structure	(123)
	LAV		Involved in modulation of NET defense through their nuclease activity	(124)
	Surface- lipoproteins		Assess in innate immune modulation and showed their nuclease activity	(125)
	NucA and NucD	*P. intermedia*	Able to degrade the DNA matrix comprising NETs	(126)
	Nuc	*A. hydrophila*	Use their nuclease to degrade NETs	(127)
	Nuc MAP3916c	*M. avium* subsp. *paratuberculosis*	Relevant to NETs degradation	(128)
	MnuA	*M. bovis*	A major membrane nuclease is rapidly degrade NETs, and play a significant role in virulence	(129)
	MbovNase		Secretory and membrane protein with ability to degrade NETs and induce apoptosis	(130)
	Mpn491	*M. pneumoniae*	Play a critical role in degradation of NETs	(131)
	Mhp597	*M. hypopneumoniae*	Involve in cytotoxicity, inflammation and degradation of NETs	(132)
	Nuc	*M. hominis*	Promoting degradation of NETs	(133)
	PAD4	*P. gingivalis*	Citrullinate the histone H3, and promoting the bacterial escape from NETs	(134)
	Gingipains		Cleavage of protease activated receptor on the neutrophil surface and prevent *P. gingivalis* from NETs entrapment	(51)
	?	*K. pneumoniae*	Affecting the mobilization of the primary granules that necessary for NETs formation	(135)

*S. aureus*, *Staphylococcus aureus*; *P. aeruginosa*, *Pseudomonas aeruginosa*; GAS, group A *Streptococcus*; *S. pneumoniae*; *Streptococcus pneumoniae*; *S. suis*, *Streptococcus suis*; *S. sanguinis*, *Streptococcus sanguinis*; *N. gonorrhoeae*, *Neisseria gonorrhoeae*; *Y. enterocolitica*; *Yersinia enterocolitica*; *V. cholera*, *Vibrio cholera*; *P. intermedia*; *Prevotella intermedia*; *M. bovis*, *Mycoplasma bovis*; *M. pneumoniae*, *Mycoplasma pneumoniae*; *M. hypopneumoniae*, *Mycoplasma hypopneumoniae*; *M. hominis*, *Mycoplasma hominis*; *P. gingivalis*, *Porphyromonas gingivalis*; *K. pneumoniae*, *Klebsiella pneumoniae*; Nuc, nuclease; Eap, extracellular adhesion protein; Ads, adenosine synthase; FnBPB, fibronectin-binding protein B; EddB, extracellular DNA degradation protein B; EddA; extracellular DNA degradation protein A; PAD4; protein arginine deiminase 4; SpnA, *Streptococcus pyogenes* nuclease A; EndA, endonuclease A; SsnA, *Streptococcus suis* nuclease A; SWAN, streptococcal wall-anchored nuclease; LAV, variable region of *Leptospira* immunoglobulin-like protein A; MnuA, membrane nuclease A; MbovNase, *Mycoplasma bovis* nuclease; Mpn491, *Mycoplasma pneumoniae* nuclease 491; Mhp597, *Mycoplasma hypopneumoniae* 597; ?, unknown.

#### 4.2.1 Nuclease expression

DNA forms a NET framework that holds multiple enzymes in close proximity, sometimes allowing for synergistic interactions among them (68). DNA is hydrolyzed by nucleases that belong to a group of hydrolases, which are further classified as endonucleases and exonucleases. These enzymes are involve in replication or repair of genetic material to maintain chromosome (136). However, extracellular nucleases have been reported in some bacterial species for the degradation of NET scaffold DNA, enabling bacteria to evade the NET antimicrobial mechanism, promoting pathogenicity, and dissemination to other sites in the host. For example, nuclease (Nuc) (101, 102) and extracellular adherence protein (Eap) (103, 104) are produced by *S. aureus* and interfere with the antimicrobial activity of NETs. These proteins help *S. aureus* to escape from NET-mediated killing, impede its removal, and increase infection-related mortality. Moreover, *S. aureus* escapes this defense by converting NETs to deoxyadenosine (dAdo) via the action of Nuc and adenosine synthase A (AdsA), which triggers caspase-3-mediated death of macrophages (105, 106). Moreover, *S. aureus* synchronizes gene expression during skin infection through the ArlRS-MgrA regulatory system, which regulates nuclease expression. This cascade is required for both the appropriate structuring of the abscess and evasion of the host innate immune system, both of which are necessary for *S. aureus* virulence. In contrast, mutants lacking MgrA and ArlRS have reduced capacity to avoid NET function (107). In addition*, S. aureus* expresses the competence regulator (ComK) when exposed to ROS. ComK upregulates the expression of genes encoding the transport machinery for glucose and DNA uptake, providing extra nutrients to increase the fermentation possibility of bacteria that are unable to respire and a source of nucleotides to repair DNA damage from ROS. Bacteria may use competence-related genes to better withstand NETs, because NETs are a source of both nucleotides and ROS (108).

*P. aeruginosa* encodes an operon of two secreted enzymes, a DNase and a predicted alkaline phosphatase. DNase (*eddB*) contributes to the degradation of NET to defend *P. aeruginosa* against NET-mediated killing, whereas *eddA* has both alkaline phosphatase and phosphodiesterase (PDase) activities that do not cause DNA degradation similar to that of DNase, but its protective function is likely a result of removing cation-chelating phosphates from the extracellular DNA phosphodiester backbone (110).

GAS is a leading cause of severe invasive disease in humans, and has evolved numerous virulence factors that aid in blocking NET function through the expression and secretion of the extracellular nuclease Sda1, which is advantageous for promoting bacterial dissemination throughout the host organism and evasion of the host innate immune response (77, 112). Similarly, *S. pyogenes* nuclease A (SpnA), a cell wall-anchored DNase, shows a unique protein architecture and promotes survival in human blood and in neutrophil killing assays, enable for the destruction of NETs and is believed to be an important immune evasion mechanism (113). A pneumococcal nuclease (EndA) acts as a virulence determinant, counteracting host-mediated trapping by NETs, thereby promoting bacterial spread from local sites to the lungs and then to the blood stream (76). Furthermore, the competence-independent activity of EndA is important for the virulence of *Streptococcus pneumoniae*, which mediates the rapid degradation of extracellular DNA and NETs (114). In addition, the secretion of nucleotide sequence-independent endodeoxyribonuclease TatD from *S. pneumoniae* is a novel potential extracellular DNase that plays a key role in evading NET-mediated bactericidal activity (115).

*Streptococcus suis* induces NET formation in porcine neutrophils and is entrapped, but not killed by NETs. The amount of NETs reduces over time due to the expression of nuclease A (SsnA), indicating that SsnA is a specific NET evasion factor in *S. suis* (116). In this sense, NET degradation is mediated not only by the known secreted SsnA but also by a putative endonuclease A of *S. suis* that is homologous to pneumococcal EndA (117). *Actinobacillus pleuropneumoniae* does not produce its own NET-degrading nucleases, but is hijacking other nucleases from host or from other co-infecting bacteria such as *S. suis* as a source for nicotinamide adenine dinucleotide (NAD) needed for efficient growth in the presence of NETs (137).

Moreover, *Streptococcus sanguinis* utilizes cell surface nuclease with a cell-wall anchor domain, termed streptococcal wall-anchored nuclease (SWAN), and contributes to bacterial resistance against the bactericidal activity of NETs (119). *Neisseria gonorrhoeae* encodes a putatively secreted thermonuclease that is implicated in biofilm remodeling and degrades the NET matrix to help *N. gonorrhoeae* from killing by neutrophils (120).

*Yersinia enterocolitica* O:3, O:8 and O:9 are able to induce NETs in human blood-derived neutrophils, but the amount of NETs is reduced at a later time, suggesting that the degradation of NETs has occurred and postulates that *Y. enterocolitica* produces Ca^2+^/Mg^2+^-dependent NET-degrading nuclease (121). *Vibrio cholera* induces the formation of NETs upon contact with neutrophils, while *V. cholerae* in return to expresses two extracellular nucleases, Dns and Xds, in the presence of NETs, and rapidly degrades the DNA component of the NETs by the combined activity of the two nucleases (122). *Leptospira* spp. is able to induce NETs using human *ex vivo* and murine *in vivo* models, resulting in the release of NETs. However, *Leptospira* spp. exerts nuclease activity and degrades DNA, resulting in a significantly reduced amount of NETs (123). In addition, the domain of the variable region of *Leptospira* immunoglobulin-like protein A (LAV) is involved in immune modulation; LAV has a nuclease activity and is demonstrated in the evasion of *Leptospira* from NETs (124). Additionally, surface-exposed lipoproteins in *Leptospira* are important for modulating host immune responses, and most of Len family surface proteins exhibit nuclease activity and are linked to NETs degradation (125).

Oral periodontopathogenic *Prevotella intermedia* produces two nucleases, NucA and NucD, which require Mg^2+^ and Ca^2+^ for their nuclease activity and contribute to NETs degradation (126). The fish pathogen *Aeromonas hydrophila* combats NETs using nuclease activity, while treatment of cells with β-glucan significantly protects NETs against bacterial degradation (127). *Mycobacterium avium* subsp. *paratuberculosis* encodes an extracellular nonspecific DNase that can destroy the NETs and promotes bacterial survival *in vitro* and *in vivo* (128).

*Mycoplasma* are the smallest bacteria that can infect and cause serious disorders in humans and various animal species (138, 139). Nucleases are a crucial component of *Mycoplasma* pathogenesis, facilitating bacterial growth and persistence in the host by digesting host nucleic acids and producing free nucleotide precursors (140–142). Membrane-associated or secreted nucleases have been found in many *Mycoplasma* species (129, 132, 143–146), and are homologous to staphylococcal nuclease (101). *Mycoplasma* lipoproteins are a major determinants of NET release during the innate immune response (39). Interestingly, most of the described *Mycoplasma* nucleases are able to escape from NET entrapment and killing by digesting NET’s DNA backbone, reducing structure stability, and enhancing NET elimination (129–131, 133).

#### 4.2.2 PAD4 secretion

Although DNA has been thoroughly examined, it is not the only NET component that is susceptible to degradation. PAD4 capable of deaminating arginine to citrulline and has been linked to NET formation in various context (147). PAD4 is involved in NET-mediated bacterial trapping and killing. Histones with cationic residues interact with negatively charged bacterial membranes and disrupt them (148). Thus, the loss of the positive charge of histones can lower their antibacterial function due to PAD4 activity. For example, *Porphyromonas gingivalis* produces its own PAD, *Porphyromonas* peptidylarginine deiminase (PPAD), which citrullinates histone H3, thereby facilitating bacterial escape from NETs, whereas PAD-mutant *P. gingivalis* is more prone to NET-mediated killing than its wild-type counterpart (134). Moreover, histone 3.1 displays bactericidal activity against *P. aeruginosa*. This bactericidal effect is reduced following citrullination by PAD4 or proteolysis by NE (111).

#### 4.2.3 Degradation of NET-bound proteins

As previously mentioned, host defense peptides are key components of the innate immune system and act as the principal first line of defense against invading pathogens (149). The cysteine protease ApdS from *S. suis* cleaves cathelicidin LL-37 and impairs its ability to promote NETs formation and ROS production (118). *K. pneumoniae* affects the mobilization of primary granules and their components, which harbor proteins with more potent bactericidal properties and those related to NETs (135). Gingipain is a cysteine protease responsible for the virulence of *P. gingivalis* and is dependent on proteolytic activation of protease-activated receptor-2 (PAR-2). Intriguingly, *P. gingivalis* and purified Arg-specific gingipains (Rgp) induce NETs that not only lack bactericidal activity, but also stimulate the growth of bacterial species otherwise susceptible to killing in NETs. Taken together, gingipains play a dual role in NETs; they are potent direct inducers of NETs formation; however, their activity prevents *P. gingivalis* entrapment and subsequent killing (51). *S. aureus* produces a cell-wall-anchored protein known as fibronectin-binding protein B (FnBPB) that is the main histone receptor and bind to all types of histones. FnBPB provides a dual immune-evasion function that captures histones, and prevents them from reaching the bacterial membrane, and simultaneously binds plasminogen, thereby promoting its conversion to plasmin to destroy the bound histone (109).

### 4.3 NET resistance mechanisms

In addition to inhibition of NET release and the degradation of NETs components, certain bacteria are resistant to the antimicrobial activity of NETs (**Table 3**).

**Table 3:** Molecules involved in the resistance of NET components in bacterial infections.

Evasion strategy	Molecule (s)	Microorganism	Modulatory effect on NETs formation	References
Resistance	D-alanylated LTA	*S. pneumoniae*	Mediates the incorporation of d-alanine residues into LTA, introducing positive charge, and reduces NETs trapping	(150)
	Biofilm		Provide resistance to NET-mediated killing	(151)
	Lipid A of LPS	*N. meningitidis*	Phosphoethanolamine modification of LPS interfere with the action of NET-bound cathepsin G	(75)
	ZnuD		Essential for absorbing Zn^2+^ and reduces the effects of nutritional immunity mediated by NETs	(75)
	OMVs		Suppressing adherence to NETs	(75)
	CPS	*K. kingae*	Protecting from ROS-mediated killing and antimicrobial peptides	(152)
	M1	GAS	Provides resistance to NETs-derived cathelicidin LL-37 by sequestering the cationic peptides	(15, 153)
			Provide resistance against NETs-mediated histones	(14)
	T4 pili	GAS M4	Sequester haptoglobin to confer M4 GAS resistance to antibacterial of LL-37 and CRAMP	(154)
	Hyaluronic acid capsule	GAS M1T1	Promote resistance to human cathelicidin LL-37 resistance and survival within NETs	(155)
	M protein			
	Collagen-like protein-1		Inhibit NET-released MPO and protects GAS from antimicrobial peptides within the NETs	(156)
	PVL and γ-hemolysin AB	*S. aureus*	Necessary for biofilm-mediated neutrophil killing	(12)
	LOS	NTHI	Initiate in the development of biofilm and mediate resistance to NET killing	(23, 157)
	Peroxiredoxin-glutaredoxin and catalase		Inhibit the oxidative burst in NETs	(158)
	Exopolysaccharide,T3SS	*P. aeruginosa*	Formation of biofilm and dead zone and display resistant to neutrophil killing	(71)
	Exopolysaccharide		Acquire resistance to NET-mediated killing in the CF airway	(74)
	OMVs		Inhibition adherence to NETs	(159)
	Biofilm	*S. suis*	Inhibit the formation of NETs	(160)

*S. pneumoniae*, *Streptococcus pneumoniae*; *N. meningitides*, *Neisseria meningitidis*; *K. kingae*, *Kingella kingae*; GAS, group A *Streptococcus*; NTHI, nontypeable *Haemophilus influenzae*; *S. aureus*, *Staphylococcus aureus*; *Pseudomonas aeruginosa*, *P. aeruginosa*; *S. suis*, *Streptococcus suis*; LTA, lipoteichoic acid; LPS, lipopolysaccharide; OMVs, outer membrane vesicles; CPS, capsular polysaccharide; PVL, Panton Valentine leucocidin; CRAMP, cathelicidin-related antimicrobial peptide; LOS, lipooligosaccharide; MPO, myeloperoxidase; T3SS, type 3 protein secretion system.

#### 4.3.1 Capsule

Capsule is a surface structure of the organism and plays a critical role in virulence, principally by interfering with host clearance mechanisms. The thickness of the pneumococcal capsule plays a crucial role in determining the extent of NET formation and may contribute to pneumonia severity (161, 162). Interestingly, the electrostatic charge of the capsule helps evade NET-mediated killing by repelling interactions with antimicrobial proteins in the DNA framework. In this context, *S. pneumoniae* contains a *dlt* operon that mediates the incorporation of d-alanine residues into lipoteichoic acid (LTA), thereby introducing a positive charge that reduces trapping by NETs *in vitro* (150). *Kingella kingae* polysaccharide capsule and exopolysaccharide function distinctly to promote neutrophil evasion. The *K. kingae* polysaccharide capsule prevents ROS production and neutrophil association, whereas the *K. kingae* exopolysaccharide reduces neutrophil phagocytosis and sensitivity to antimicrobial peptides (152). In addition, the hyaluronic acid capsule and M protein of the GAS serotype M1T1 promote resistance to human cathelicidin LL-37, which may be an important contributor to the NET-resistance phenotype (155).

#### 4.3.2 Biofilm formation

Microbial biofilm communities are microbes embedded in a matrix of self-produced polysaccharides and other molecules, such as lipids, proteins, and nucleic acids that can bind to various surfaces, which complicates the immunological and therapeutic response (163). Biofilm formation by *P. aeruginosa* is a major cause of bacterial keratitis and is facilitated by bacterial Psl exopolysaccharide and T3SS. NETs are stimulated by high expression of T3SS and form a barrier, known as dead-zone, confining bacteria to the external corneal environment, preventing them from spreading to the brain. Once formed, ocular biofilms advance eye pathology because they are resistant to neutrophil killing and antibiotics (71). Furthermore, *P. aeruginosa* acquires resistance to NET-mediated killing in cystic fibrosis airways. This resistance correlates with the development of excess exopolysaccharide production (which characterizes the mucoid phenotype) (74). In addition, studies conducted *in vitro* have demonstrated that *P. aeruginosa* can profit from the released DNA of NETs and incorporate it into the extracellular matrix, potentially strengthening the matrix’s resistance to antibiotics and host antimicrobial peptides (164–167).

Clinical isolates of *H. influenzae* share a common tendency to produce biofilms, and the factors that facilitate biofilm formation include the expression of certain lipooligosaccharide (LOS) glycoforms (168–170), type IV pili (171), and double-stranded DNA release (172). Nontypeable *Haemophilus influenzae* (NTHi) promotes resistance to killing within NETs structures through LOS moieties that promote biofilm formation (23, 157). Methicillin-resistant *S. aureus* (MRSA) biofilms rapidly skew neutrophils toward NETs formation through the combined activity of the Panton-Valentine leukocidin (PVL) and γ-hemolysin AB, which are important for biofilm-mediated neutrophil killing. Through this response, *S. aureus* can persist because the antimicrobial activity of the released NETs is ineffective in eradicating bacterial biofilm (12). *S. aureus* nuclease-mediated NET degradation promotes the persistence of biofilm bacteria entrapped in NETs (173). *S. suis* serotype 2 induces NETs release and can be captured by the NETs, whereas biofilm formation inhibit NETs release through the biofilm extracellular matrix and enables survival by allowing the pathogen to persist and resist the host immune system (160). In addition, pneumococci produce biofilms *in vivo* and observe web-or net-like structures surrounded by dense matrix material, which is intertwined with the formation of NETs (151).

#### 4.3.3 Outer membrane vesicles

OMVs are nanostructures released by pathogenic and non-pathogenic bacteria *in vivo* and *in vitro* and can act as decoys for NET capture (174). Ocular keratitis is often associated with *P. aeruginosa* infection. Neutrophils release NETs in response to both cytotoxic and invasive clinical isolates of *P. aeruginosa*. Cytotoxic strains are less prone to NET capture than invasive strains because they release OMVs that inhibit NET adherence (159). Furthermore, *Neisseria meningitidis* releases OMVs as potent NET inducers. NETs are unable to kill NET- bound meningococci, but they slow down their proliferation rate. The bacteriostatic effect of NETs is counteracted by spontaneously released OMVs from *N. meningitidis*, which reduces their adherence to NETs (75).

#### 4.3.4 Charge surface alteration

NET-releasing proteins attach to negatively charged phospholipids in pathogen membranes with electrostatic affinity, which promotes death (175). Nonetheless, certain bacteria modify their cell surfaces, which reduces their affinity for attachment to NET-releasing peptides. For example, the surface-associated protein M1 protein contributes to GAS virulence by interfering with NET-mediated killing, which sequester and neutralize cathelicidin LL-37 through the N-terminal hypervariable (HV) region and A repeat region that encode the type-specific immunologic epitopes of the M1 serotype (15, 153). In addition, the N-terminal portion of the M1 protein binds and inactivates histones before they reach their cell wall target of action and mediate resistance against released extracellular histones in NETs, allowing the pathogen to tolerate high concentrations of histones and promote survival in NETs (14). Streptococcal collagen-like protein 1 (Scl-1) in GAS serotype M1T1 confers resistance to NET-mediated killing, which may be in large part due to the antimicrobial peptides present within the NETs; Scl-1 has an additional role in suppressing the release of MPO, which ultimately limits the production of NETs (156). Haptoglobin is an abundant acute-phase protein produced upon infection, which binds to human neutrophils and monocytes and inhibits their functions (176, 177). The T4 antigen, the pilus backbone protein of GAS M4, sequester the host haptoglobin. Coating M4 GAS with haptoglobin causes a reduction in susceptibility to cathelicidin LL-37 and murine cathelicidin-related antimicrobial peptide (CRAMP) and promotes resistance to NET-mediated killing (154).

Modification of lipid A of meningococcal LPS with phosphoethanolamine shields *N. meningitidis* from the action of NET-bound cathepsin G (75). In a process known as nutritional immunity, host organisms restrict the availability of trace nutrients in the blood or secretions to prevent the growth of invading microorganisms (178). The outer membrane receptor ZnuD of *N. meningitidis* is crucial for Zn^2+^ uptake at very low concentrations (179). *N. meningitidis* utilizes high affinity absorption systems for critical ions on mucosal surfaces, producing ZnuD, which absorbs Zn^2+^; contribute to survival within NETs (75).

#### 4.3.5 Antioxidant enzymes secretion

Finally, the initiation of the oxidative burst is important for the induction of NETs, and oxidants contribute to microbicidal activity within the NETs (9, 180). Noticeable *H. influenzae* expresses the bifunctional peroxiredoxin-glutaredoxin (encoded by *pdgX*) and catalase (encoded by *hktE*), which confer resistance of NTHI to oxidative killing and thus promote the survival of NTHI within NET structures and persistence *in vivo* in the lung and middle ear. In addition, exogenous catalase partially rescued NTHI from NET-mediated killing *in vitro.* The expression of both peroxiredoxin-glutaredoxin and catalase is a mechanism by which NTHI combats the effects of NETs (158).

## 5 Concluding remarks

Neutrophils are type of white blood cell that play a crucial role in the immune system’s defense against infections. There are different subsets of neutrophils that have different functions and responses to various stimuli. NET release is a form of neutrophil immune response that, depending on the context in which it is examined, can have both pathogenic and physiological effects. They can entangle and immobilize bacteria, preventing their spread, and facilitating their destruction by other immune cells. Additionally, dysregulation of NET release has several implications for the immune system and overall health. On the other hand, impaired NET formation may lead to reduced ability to fight off infections. Therefore, maintaining a balanced and controlled NET release is essential for the proper functioning of the defense system and overall health. In recent years, there has been a significant increase in research focused on the role NETs in response to pathogens and the mechanisms involved in the modulation of their release. Bacteria have developed various mechanisms to evade or resist NET release, allowing them to escape the immune system’s defenses and several key findings and advancements have emerged from these studies. In this review, we have discussed the strategies used by various bacteria to counteract NET-mediated antimicrobial effects as such inhibiting NET release, deactivating their components, degrading their net-like framework, or blocking their contact, resulting in infection dissemination and immune system inactivation. Bacteria possess a vast array of mechanism for modulating NET activity, and ongoing investigations are expected to uncover new molecules and pathways involved in controlling NET release. Current understanding of different inflammatory mediators in modulate NET release is quite limited and need be further elucidated. Overall, the recent focus on NETs and their modulation has provided a deeper understanding of the complex interplay between neutrophils, pathogens, and the immune system. This knowledge has the potential to identify pharmacological targets and drive the development of therapeutic and diagnostic approaches to counteract bacterial evasion strategies and combat the role of NETs in inflammatory and autoimmune diseases. It is crucial to understand that various factors affect whether NETs are advantageous or harmful, with the dose and the timing of NET release and clearance being critical factors. A better understanding of the roles of NETs and their effects on hosts will make it possible to inhibit the adverse attributes without affecting the beneficial ones, which will ultimately enable strategies related to NETs to be used in disease treatment.

## Author contributions

AA: Investigation, Visualization, Writing – original draft. HH: Investigation, Visualization, Writing – review & editing. SLan: Investigation, Visualization, Writing – review & editing. ZL: Investigation, Visualization, Writing – review & editing. SLiu: Investigation, Visualization, Writing – review & editing. SC: Conceptualization, Funding acquisition, Investigation, Supervision, Visualization, Writing – review & editing. YC: Funding acquisition, Project administration, Supervision, Writing – review & editing.

## Glossary

**Table:**

ACT	Adenylate cyclase toxin
AdsA	Adenosine synthase A
C5a	Complement factor 5a
cAMP	Cyclic adenosine monophosphate
CLRs	C-type lectin receptors
ComK	Competence regulator
CPS-I	Capsular polysaccharide I
CRAMP	Cathelicidin-related antimicrobial peptide
CRs	Complement receptors
dAdo	Deoxyadenosine
Eap	Extracellular adherence protein
FnBPB	Fibronectin-binding protein B
GAS	Group A *Streptococcus*
GBS	Group B *Streptococcus*
GM-CSF	Granulocyte-macrophage colony-stimulating factor
HMW-HA	High-molecular-weight hyaluronic acid
IFN	Interferon
IL	Interleukin
LOS	Lipooligosaccharide
LPS	Lipopolysaccharide
LTA	Lipoteichoic acid
LTH	Leukotoxic hypercitrullination
MAPK	Mitogen-activated protein kinase
MPO	Myeloperoxidase
MRSA	Methicillin-resistant *S. aureus*
NE	Neutrophil elastase
NETs	Neutrophil extracellular traps
NLRs	Nucleotide-binding oligomerization domain-like receptors
NTHi	Nontypeable *Haemophilus influenzae*
Nuc	Nuclease
OMVs	Outer membrane vesicles
PAD4	Protein arginine deiminase 4
PAR-2	Protease activated receptor-2
PVL	Panton-Valentine leukocidin
ROS	Reactive oxygen species
Scl-1	Streptococcal collagen-like protein 1
Siglec-5	Sialic acid-binding Ig-like lectin-5
Siglec-9	Sialic acid-binding Ig-like lectin-9
SLO	Streptolysin O toxin
SWAN	Streptococcal wall-anchored nuclease
T3SS	Type 3 protein secretion system
TGF	Transforming growth factor
TLRs	Toll-like receptors
TNF	Tumor necrosis factor
Wip1	Wild-type p53-induced phosphatase 1

## References

[1] Gierlikowska B Stachura A Gierlikowski W Demkow U. Phagocytosis, degranulation and extracellular traps release by neutrophils-the current knowledge, pharmacological modulation and future prospects. Front Pharmacol (2021) 12:666732. doi: 10.3389/fphar.2021.666732 34017259 PMC8129565

[2] Schumann J. It is all about fluidity: Fatty acids and macrophage phagocytosis. Eur J Pharmacol (2016) 785(15):18–23. doi: 10.1016/j.ejphar.2015.04.057 25987422

[3] Jaumouillé V Waterman CM. Physical constraints and forces involved in phagocytosis. Front Immunol (2020) 11:1097. doi: 10.3389/fimmu.2020.01097 32595635 PMC7304309

[4] Mortaz E Alipoor SD Adcock IM Mumby S Koenderman L. Update on neutrophil function in severe inflammation. Front Immunol (2018) 9:2171. doi: 10.3389/fimmu.2018.02171 30356867 PMC6190891

[5] Kolaczkowska E Kubes P. Neutrophil recruitment and function in health and inflammation. Nat Rev Immunol (2013) 13(3):159–75. doi: 10.1038/nri3399 23435331

[6] Faurschou M Borregaard N. Neutrophil granules and secretory vesicles in inflammation. Microbes infection. (2003) 5(14):1317–27. doi: 10.1016/j.micinf.2003.09.008 14613775

[7] Brinkmann V Reichard U Goosmann C Fauler B Uhlemann Y Weiss DS. Neutrophil extracellular traps kill bacteria. Science. (2004) 303(5663):1532–5. doi: 10.1126/science.1092385 15001782

[8] Manda A Pruchniak MP Araźna M Demkow UA. Neutrophil extracellular traps in physiology and pathology. Central-European J Immunol (2014) 39(1):116–21. doi: 10.5114/ceji.2014.42136 PMC4439979 26155111

[9] Fuchs TA Abed U Goosmann C Hurwitz R Schulze I Wahn V. Novel cell death program leads to neutrophil extracellular traps. J Cell Biol (2007) 176(2):231–41. doi: 10.1083/jcb.200606027 PMC2063942 17210947

[10] Yipp BG Kubes P. NETosis: How vital is it? Blood (2013) 122(16):2784–94. doi: 10.1182/blood-2013-04-457671 24009232

[11] Zawrotniak M Rapala-Kozik M. Neutrophil extracellular traps (NETs) - formation and implications. Acta Biochim Polonica. (2013) 60(3):277–84. doi: 10.18388/abp.2013_1983 23819131

[12] Bhattacharya M Berends ETM Chan R Schwab E Roy S Sen CK. *Staphylococcus aureus* biofilms release leukocidins to elicit extracellular trap formation and evade neutrophil-mediated killing. Proc Natl Acad Sci U S A. (2018) 115(28):7416–21. doi: 10.1073/pnas.1721949115 PMC6048508 29941565

[13] Pilsczek FH Salina D Poon KK Fahey C Yipp BG Sibley CD. A novel mechanism of rapid nuclear neutrophil extracellular trap formation in response to Staphylococcus aureus. J Immunol (2010) 185(12):7413–25. doi: 10.4049/jimmunol.1000675 21098229

[14] Döhrmann S LaRock CN Anderson EL Cole JN Ryali B Stewart C. Group A streptococcal M1 protein provides resistance against the antimicrobial activity of histones. Sci Rep (2017) 7:43039. doi: 10.1038/srep43039 28220899 PMC5318940

[15] Lauth X von Köckritz-Blickwede M McNamara CW Myskowski S Zinkernagel AS Beall B. M1 protein allows Group A streptococcal survival in phagocyte extracellular traps through cathelicidin inhibition. J innate immunity. (2009) 1(3):202–14. doi: 10.1159/000203645 PMC3241932 20375578

[16] Mori Y Yamaguchi M Terao Y Hamada S Ooshima T Kawabata S. α-Enolase of *Streptococcus pneumoniae* induces formation of neutrophil extracellular traps. J Biol Chem (2012) 287(13):10472–81. doi: 10.1074/jbc.M111.280321 PMC3323051 22262863

[17] Kenny EF Herzig A Krüger R Muth A Mondal S Thompson PR. Diverse stimuli engage different neutrophil extracellular trap pathways. Elife. (2017) 6:e24437. doi: 10.7554/eLife.24437 28574339 PMC5496738

[18] Sumioka R Nakata M Okahashi N Li Y Wada S Yamaguchi M. *Streptococcus sanguinis* induces neutrophil cell death by production of hydrogen peroxide. PloS One (2017) 12(2):e0172223. doi: 10.1371/journal.pone.0172223 28222125 PMC5319702

[19] Dai J Lai L Tang H Wang W Wang S Lu C. *Streptococcus suis* synthesizes deoxyadenosine and adenosine by 5'-nucleotidase to dampen host immune responses. Virulence. (2018) 9(1):1509–20. doi: 10.1080/21505594.2018.1520544 PMC6177238 30221577

[20] Pieterse E Rother N Yanginlar C Hilbrands LB van der Vlag J. Neutrophils discriminate between lipopolysaccharides of different bacterial sources and selectively release neutrophil extracellular traps. Front Immunol (2016) 7:484. doi: 10.3389/fimmu.2016.00484 27867387 PMC5095130

[21] Grinberg N Elazar S Rosenshine I Shpigel NY. Beta-hydroxybutyrate abrogates formation of bovine neutrophil extracellular traps and bactericidal activity against mammary pathogenic Escherichia coli. Infection Immun (2008) 76(6):2802–7. doi: 10.1128/IAI.00051-08 PMC2423099 18411287

[22] Krivošíková K Šupčíková N Gaál Kovalčíková A Janko J Pastorek M Celec P. Neutrophil extracellular traps in urinary tract infection. Front pediatrics. (2023) 11:1154139. doi: 10.3389/fped.2023.1154139 PMC10067609 37020646

[23] Hong W Juneau RA Pang B Swords WE. Survival of bacterial biofilms within neutrophil extracellular traps promotes nontypeable *Haemophilus influenzae* persistence in the chinchilla model for otitis media. J innate immunity. (2009) 1(3):215–24. doi: 10.1159/000205937 PMC6951045 20375579

[24] Rada B Jendrysik MA Pang L Hayes CP Yoo DG Park JJ. Pyocyanin-enhanced neutrophil extracellular trap formation requires the NADPH oxidase. PloS One (2013) 8(1):e54205. doi: 10.1371/journal.pone.0054205 23342104 PMC3544820

[25] Floyd M Winn M Cullen C. Swimming motility mediates the formation of neutrophil extracellular traps induced by flagellated pseudomonas aeruginosa. PloS Pathog (2016) 12(11):e1005987. doi: 10.1371/journal.ppat.1005987 27855208 PMC5113990

[26] Gillenius E Urban CF. The adhesive protein invasin of *Yersinia pseudotuberculosis* induces neutrophil extracellular traps via β1 integrins. Microbes infection. (2015) 17(5):327–36. doi: 10.1016/j.micinf.2014.12.014 25576025

[27] Altincicek B Stötzel S Wygrecka M Preissner KT Vilcinskas A. Host-derived extracellular nucleic acids enhance innate immune responses, induce coagulation, and prolong survival upon infection in insects. J Immunol (2008) 181(4):2705–12. doi: 10.4049/jimmunol.181.4.2705 18684961

[28] Ramos-Kichik V Mondragón-Flores R Mondragón-Castelán M Gonzalez-Pozos S Muñiz-Hernandez S Rojas-Espinosa O. Neutrophil extracellular traps are induced by Mycobacterium tuberculosis. Tuberculosis (2009) 89(1):29–37. doi: 10.1016/j.tube.2008.09.009 19056316

[29] Braian C Hogea V Stendahl O. *Mycobacterium tuberculosis*- induced neutrophil extracellular traps activate human macrophages. J innate immunity. (2013) 5(6):591–602. doi: 10.1159/000348676 23635526 PMC6741595

[30] Ladero-Auñon I Molina E Holder A Kolakowski J Harris H Urkitza A. Bovine Neutrophils Release Extracellular Traps and Cooperate With Macrophages in *Mycobacterium avium* subsp. paratuberculosis clearance In Vitro. Front Immunol (2021) 12:645304. doi: 10.3389/fimmu.2021.645304 33815401 PMC8010319

[31] Gondaira S Nishi K Fujiki J Iwano H Watanabe R Eguchi A. Innate immune response in bovine neutrophils stimulated with Mycoplasma bovis. Vet Res (2021) 52(1):58. doi: 10.1186/s13567-021-00920-2 33863386 PMC8052696

[32] Martinelli S Urosevic M Daryadel A Oberholzer PA Baumann C Fey MF. Induction of genes mediating interferon-dependent extracellular trap formation during neutrophil differentiation. J Biol Chem (2004) 279(42):44123–32. doi: 10.1074/jbc.M405883200 15302890

[33] Keshari RS Jyoti A Dubey M Kothari N Kohli M Bogra J. Cytokines induced neutrophil extracellular traps formation: implication for the inflammatory disease condition. PloS One (2012) 7(10):e48111. doi: 10.1371/journal.pone.0048111 23110185 PMC3482178

[34] Long J Sun Y Liu S Yang S Chen C Zhang Z. Targeting pyroptosis as a preventive and therapeutic approach for stroke. Cell Death discovery. (2023) 9(1):155. doi: 10.1038/s41420-023-01440-y 37165005 PMC10172388

[35] Lv M Wang Y Yu J Kong Y Zhou H Zhang A. Grass carp Il-2 promotes neutrophil extracellular traps formation via inducing ROS production and autophagy in vitro. Fish shellfish Immunol (2024) 144:109261. doi: 10.1016/j.fsi.2023.109261 38040137

[36] Mikhalchik E Basyreva LY Gusev SA Panasenko OM Klinov DV Barinov NA. Activation of neutrophils by mucin-vaterite microparticles. Int J Mol Sci (2022) 23(18):10579. doi: 10.3390/ijms231810579 36142492 PMC9501559

[37] Wang Y Li M Stadler S Correll S Li P Wang D. Histone hypercitrullination mediates chromatin decondensation and neutrophil extracellular trap formation. J Cell Biol (2009) 184(2):205–13. doi: 10.1083/jcb.200806072 PMC2654299 19153223

[38] Yousefi S Mihalache C Kozlowski E Schmid I Simon HU. Viable neutrophils release mitochondrial DNA to form neutrophil extracellular traps. Cell Death differentiation. (2009) 16(11):1438–44. doi: 10.1038/cdd.2009.96 19609275

[39] Cacciotto C Cubeddu T Addis MF Anfossi AG Tedde V Tore G. Mycoplasma lipoproteins are major determinants of neutrophil extracellular trap formation. Cell Microbiol (2016) 18(12):1751–62. doi: 10.1111/cmi.12613 27166588

[40] Ma F Chang X Wang G Zhou H Ma Z Lin H. *Streptococcus Suis* Serotype 2 Stimulates Neutrophil Extracellular Traps Formation via Activation of p38 MAPK and ERK1/2. Front Immunol (2018) 9:2854. doi: 10.3389/fimmu.2018.02854 30581435 PMC6292872

[41] Tamarozzi F Turner JD Pionnier N Midgley A Guimaraes AF Johnston KL. *Wolbachia endosymbionts* induce neutrophil extracellular trap formation in human onchocerciasis. Sci Rep (2016) 6:35559. doi: 10.1038/srep35559 27752109 PMC5067710

[42] Clark SR Ma AC Tavener SA McDonald B Goodarzi Z Kelly MM. Platelet TLR4 activates neutrophil extracellular traps to ensnare bacteria in septic blood. Nat Med (2007) 13(4):463–9. doi: 10.1038/nm1565 17384648

[43] Wan T Zhao Y Fan F Hu R Jin X. Dexamethasone Inhibits S. aureus-Induced Neutrophil Extracellular Pathogen-Killing Mechanism, Possibly through Toll-Like Receptor Regulation. Front Immunol (2017) 8:60. doi: 10.3389/fimmu.2017.00060 28232829 PMC5299007

[44] Hook JS Patel PA O'Malley A Xie L Kavanaugh JS Horswill AR. Lipoproteins from *staphylococcus aureus* drive neutrophil extracellular trap formation in a TLR2/1- and PAD-dependent manner. J Immunol (2021) 207(3):966–73. doi: 10.4049/jimmunol.2100283 PMC8324556 34290104

[45] Alyami HM Finoti LS Teixeira HS Aljefri A Kinane DF Benakanakere MR. Role of NOD1/NOD2 receptors in Fusobacterium nucleatum mediated NETosis. Microbial pathogenesis. (2019) 131:53–64. doi: 10.1016/j.micpath.2019.03.036 30940608

[46] Chen ST Li FJ Hsu TY Liang SM Yeh YC Liao WY. CLEC5A is a critical receptor in innate immunity against *Listeria* infection. Nat Commun (2017) 8(1):299. doi: 10.1038/s41467-017-00356-3 28824166 PMC5563510

[47] Palmer LJ Damgaard C Holmstrup P Nielsen CH. Influence of complement on neutrophil extracellular trap release induced by bacteria. J periodontal Res (2016) 51(1):70–6. doi: 10.1111/jre.12284 25900429

[48] Alemán OR Mora N Cortes-Vieyra R Uribe-Querol E Rosales C. Differential use of human neutrophil fc receptors for inducing neutrophil extracellular trap formation. J Immunol Res (2016) 2016:2908034. doi: 10.1155/2016/2908034 27034964 PMC4806689

[49] Alemán OR Mora N Cortes-Vieyra R Uribe-Querol E Rosales C. Transforming growth factor-β-activated kinase 1 is required for human fcγRIIIb-induced neutrophil extracellular trap formation. Front Immunol (2016) 7:277. doi: 10.3389/fimmu.2016.00277 27486461 PMC4947870

[50] Diago-Navarro E Calatayud-Baselga I Sun D Khairallah C Mann I Ulacia-Hernando A. Antibody-based immunotherapy to treat and prevent infection with hypervirulent klebsiella pneumoniae. Clin Vaccine Immunol (2017) 24(1):e00456-16. doi: 10.1128/CVI.00456-16 27795303 PMC5216427

[51] Bryzek D Ciaston I Dobosz E Gasiorek A. Triggering NETosis via protease-activated receptor (PAR)-2 signaling as a mechanism of hijacking neutrophils function for pathogen benefits. PloS pathogens. (2019) 15(5):e1007773. doi: 10.1371/journal.ppat.1007773 31107907 PMC6544335

[52] Urban CF Ermert D Schmid M Abu-Abed U Goosmann C Nacken W. Neutrophil extracellular traps contain calprotectin, a cytosolic protein complex involved in host defense against Candida albicans. PloS Pathog (2009) 5(10):e1000639. doi: 10.1371/journal.ppat.1000639 19876394 PMC2763347

[53] Brinkmann V. Neutrophil extracellular traps in the second decade. J innate Immun (2018) 10(5-6):414–21. doi: 10.1159/000489829 PMC6784051 29909412

[54] Dwyer M Shan Q D'Ortona S Maurer R Mitchell R Olesen H. Cystic fibrosis sputum DNA has NETosis characteristics and neutrophil extracellular trap release is regulated by macrophage migration-inhibitory factor. J innate immunity. (2014) 6(6):765–79. doi: 10.1159/000363242 PMC4201867 24862346

[55] Hakkim A Fuchs TA Martinez NE Hess S Prinz H Zychlinsky A. Activation of the Raf-MEK-ERK pathway is required for neutrophil extracellular trap formation. Nat Chem Biol (2011) 7(2):75–7. doi: 10.1038/nchembio.496 21170021

[56] Metzler KD Goosmann C Lubojemska A Zychlinsky A Papayannopoulos V. A myeloperoxidase-containing complex regulates neutrophil elastase release and actin dynamics during NETosis. Cell Rep (2014) 8(3):883–96. doi: 10.1016/j.celrep.2014.06.044 PMC4471680 25066128

[57] Papayannopoulos V Metzler KD Hakkim A Zychlinsky A. Neutrophil elastase and myeloperoxidase regulate the formation of neutrophil extracellular traps. J Cell Biol (2010) 191(3):677–91. doi: 10.1083/jcb.201006052 PMC3003309 20974816

[58] Sollberger G Choidas A. Gasdermin D plays a vital role in the generation of neutrophil extracellular traps. Sci Immunol (2018) 3(26):eaar6689. doi: 10.1126/sciimmunol.aar6689 30143555

[59] Castanheira FVS Kubes P. Neutrophils and NETs in modulating acute and chronic inflammation. Blood. (2019) 133(20):2178–85. doi: 10.1182/blood-2018-11-844530 30898862

[60] Leshner M Wang S Lewis C Zheng H Chen XA Santy L. PAD4 mediated histone hypercitrullination induces heterochromatin decondensation and chromatin unfolding to form neutrophil extracellular trap-like structures. Front Immunol (2012) 3:307. doi: 10.3389/fimmu.2012.00307 23060885 PMC3463874

[61] Yipp BG Petri B Salina D Jenne CN Scott BN Zbytnuik LD. Infection-induced NETosis is a dynamic process involving neutrophil multitasking in vivo. Nat Med (2012) 18(9):1386–93. doi: 10.1038/nm.2847 PMC4529131 22922410

[62] Jorch SK Kubes P. An emerging role for neutrophil extracellular traps in noninfectious disease. Nat Med (2017) 23(3):279–87. doi: 10.1038/nm.4294 28267716

[63] Dunham-Snary KJ Surewaard BG Mewburn JD Bentley RE Martin AY Jones O. Mitochondria in human neutrophils mediate killing of Staphylococcus aureus. Redox Biol (2022) 49:102225. doi: 10.1016/j.redox.2021.102225 34959099 PMC8758915

[64] Konig MF Andrade F. A critical reappraisal of neutrophil extracellular traps and NETosis mimics based on differential requirements for protein citrullination. Front Immunol (2016) 7:461. doi: 10.3389/fimmu.2016.00461 27867381 PMC5095114

[65] Zhu S Yu Y Ren Y Xu L Wang H Ling X. The emerging roles of neutrophil extracellular traps in wound healing. Cell Death Dis (2021) 12(11):984. doi: 10.1038/s41419-021-04294-3 34686654 PMC8536667

[66] Tanikawa C Espinosa M Suzuki A Masuda K Yamamoto K Tsuchiya E. Regulation of histone modification and chromatin structure by the p53-PADI4 pathway. Nat Commun (2012) 3:676. doi: 10.1038/ncomms1676 22334079

[67] Daniel C Leppkes M Muñoz LE Schley G Schett G Herrmann M. Extracellular DNA traps in inflammation, injury and healing. Nat Rev Nephrology. (2019) 15(9):559–75. doi: 10.1038/s41581-019-0163-2 31213698

[68] Halverson TW Wilton M Poon KK Petri B Lewenza S. DNA is an antimicrobial component of neutrophil extracellular traps. PloS pathogens. (2015) 11(1):e1004593. doi: 10.1371/journal.ppat.1004593 25590621 PMC4295883

[69] Richards RC O'Neil DB Thibault P Ewart KV. Histone H1: an antimicrobial protein of Atlantic salmon (Salmo salar). Biochem Biophys Res Commun (2001) 284(3):549–55. doi: 10.1006/bbrc.2001.5020 11396934

[70] Weinrauch Y Drujan D Shapiro SD Weiss J Zychlinsky A. Neutrophil elastase targets virulence factors of enterobacteria. Nature. (2002) 417(6884):91–4. doi: 10.1038/417091a 12018205

[71] Thanabalasuriar A Scott BNV Peiseler M Willson ME Zeng Z Warrener P. Neutrophil extracellular traps confine *pseudomonas aeruginosa* ocular biofilms and restrict brain invasion. Cell Host Microbe (2019) 25(4):526–36.e4. doi: 10.1016/j.chom.2019.02.007 30930127 PMC7364305

[72] Marin-Esteban V Turbica I Dufour G Semiramoth N Gleizes A Gorges R. Afa/Dr diffusely adhering *Escherichia coli* strain C1845 induces neutrophil extracellular traps that kill bacteria and damage human enterocyte-like cells. Infection immunity. (2012) 80(5):1891–9. doi: 10.1128/IAI.00050-12 PMC3347451 22371374

[73] Li H Liu L Wang J Zhao W. The emerging role of neutrophil extracellular traps in endometritis. Front Immunol (2023) 14:1153851. doi: 10.3389/fimmu.2023.1153851 37033951 PMC10073465

[74] Young RL Malcolm KC Kret JE Caceres SM Poch KR Nichols DP. Neutrophil extracellular trap (NET)-mediated killing of *Pseudomonas aeruginosa*: evidence of acquired resistance within the CF airway, independent of CFTR. PloS One (2011) 6(9):e23637. doi: 10.1371/journal.pone.0023637 21909403 PMC3164657

[75] Lappann M Danhof S Guenther F Olivares-Florez S Mordhorst IL Vogel U. *In vitro* resistance mechanisms of *Neisseria meningitidis* against neutrophil extracellular traps. Mol Microbiol (2013) 89(3):433–49. doi: 10.1111/mmi.12288 23750848

[76] Beiter K Wartha F Albiger B Normark S Zychlinsky A Henriques-Normark B. An endonuclease allows *Streptococcus pneumoniae* to escape from neutrophil extracellular traps. Curr Biol (2006) 16(4):401–7. doi: 10.1016/j.cub.2006.01.056 16488875

[77] Buchanan JT Simpson AJ Aziz RK Liu GY Kristian SA Kotb M. DNase expression allows the pathogen group A *Streptococcus* to escape killing in neutrophil extracellular traps. Curr Biol (2006) 16(4):396–400. doi: 10.1016/j.cub.2005.12.039 16488874

[78] Azzouz L Cherry A Riedl M Khan M Pluthero FG Kahr WHA. Relative antibacterial functions of complement and NETs: NETs trap and complement effectively kills bacteria. Mol Immunol (2018) 97(1):71–81. doi: 10.1016/j.molimm.2018.02.019 29571059

[79] Douda DN Jackson R Grasemann H Palaniyar N. Innate immune collectin surfactant protein D simultaneously binds both neutrophil extracellular traps and carbohydrate ligands and promotes bacterial trapping. J Immunol (2011) 187(4):1856–65. doi: 10.4049/jimmunol.1004201 21724991

[80] Kolaczkowska E Jenne CN Surewaard BG Thanabalasuriar A Lee WY Sanz MJ. Molecular mechanisms of NET formation and degradation revealed by intravital imaging in the liver vasculature. Nat Commun (2015) 6:6673. doi: 10.1038/ncomms7673 25809117 PMC4389265

[81] Reeves EP Lu H Jacobs HL Messina CG Bolsover S Gabella G. Killing activity of neutrophils is mediated through activation of proteases by K+ flux. Nature. (2002) 416(6878):291–7. doi: 10.1038/416291a 11907569

[82] Mutua V Gershwin LJ. A review of neutrophil extracellular traps (NETs) in disease: Potential anti-NETs therapeutics. Clin Rev Allergy Immunol (2021) 61(2):194–211. doi: 10.1007/s12016-020-08804-7 32740860 PMC7395212

[83] Papayannopoulos V. Neutrophil extracellular traps in immunity and disease. Nat Rev Immunol (2018) 18(2):134–47. doi: 10.1038/nri.2017.105 28990587

[84] Skopelja-Gardner S Theprungsirikul J Lewis KA Hammond JH Carlson KM Hazlett HF. Regulation of *pseudomonas aeruginosa*-mediated neutrophil extracellular traps. Front Immunol (2019) 10:1670. doi: 10.3389/fimmu.2019.01670 31379861 PMC6657737

[85] Khatua B Bhattacharya K Mandal C. Sialoglycoproteins adsorbed by *Pseudomonas aeruginosa* facilitate their survival by impeding neutrophil extracellular trap through siglec-9. J leukocyte Biol (2012) 91(4):641–55. doi: 10.1189/jlb.0511260 22241833

[86] Zinkernagel AS Timmer AM Pence MA Locke JB Buchanan JT Turner CE. The IL-8 protease SpyCEP/ScpC of group A *Streptococcus* promotes resistance to neutrophil killing. Cell Host Microbe (2008) 4(2):170–8. doi: 10.1016/j.chom.2008.07.002 PMC2631432 18692776

[87] Uchiyama S Döhrmann S Timmer AM Dixit N Ghochani M Bhandari T. Streptolysin O rapidly impairs neutrophil oxidative burst and antibacterial responses to group A streptococcus. Front Immunol (2015) 6:581. doi: 10.3389/fimmu.2015.00581 26635795 PMC4644796

[88] Secundino I Lizcano A Roupé KM Wang X Cole JN Olson J. Host and pathogen hyaluronan signal through human siglec-9 to suppress neutrophil activation. J Mol Med (2016) 94(2):219–33. doi: 10.1007/s00109-015-1341-8 PMC4766071 26411873

[89] Nicchi S Giusti F Carello S Utrio Lanfaloni S Tavarini S Frigimelica E. *Moraxella catarrhalis* evades neutrophil oxidative stress responses providing a safer niche for nontypeable Haemophilus influenzae. iScience. (2022) 25(3):103931. doi: 10.1016/j.isci.2022.103931 35265810 PMC8899411

[90] Ou Q Fang JQ Zhang ZS Chi Z Fang J Xu DY. TcpC inhibits neutrophil extracellular trap formation by enhancing ubiquitination mediated degradation of peptidylarginine deiminase 4. Nat Commun (2021) 12(1):3481. doi: 10.1038/s41467-021-23881-8 34108482 PMC8190435

[91] Carlin AF Uchiyama S Chang YC Lewis AL Nizet V Varki A. Molecular mimicry of host sialylated glycans allows a bacterial pathogen to engage neutrophil Siglec-9 and dampen the innate immune response. Blood. (2009) 113(14):3333–6. doi: 10.1182/blood-2008-11-187302 PMC2665898 19196661

[92] Carlin AF Chang YC Areschoug T Lindahl G Hurtado-Ziola N King CC. Group B Streptococcus suppression of phagocyte functions by protein-mediated engagement of human Siglec-5. J Exp Med (2009) 206(8):1691–9. doi: 10.1084/jem.20090691 PMC2722167 19596804

[93] Eby JC Gray MC Hewlett EL. Cyclic AMP-mediated suppression of neutrophil extracellular trap formation and apoptosis by the *Bordetella pertussis* adenylate cyclase toxin. Infection immunity. (2014) 82(12):5256–69. doi: 10.1128/IAI.02487-14 PMC4249293 25287922

[94] Gorgojo J Scharrig E Gómez RM Harvill ET Rodríguez ME. *Bordetella parapertussis* circumvents neutrophil extracellular bactericidal mechanisms. PloS One (2017) 12(1):e0169936. doi: 10.1371/journal.pone.0169936 28095485 PMC5240980

[95] Castillo LA Birnberg-Weiss F Rodriguez-Rodrigues N Martire-Greco D Bigi F Landoni VI. *Klebsiella pneumoniae* ST258 negatively regulates the oxidative burst in human neutrophils. Front Immunol (2019) 10:929. doi: 10.3389/fimmu.2019.00929 31105712 PMC6497972

[96] Riyapa D Buddhisa S Korbsrisate S Cuccui J Wren BW Stevens MP. Neutrophil extracellular traps exhibit antibacterial activity against burkholderia pseudomallei and are influenced by bacterial and host factors. Infection immunity. (2012) 80(11):3921–9. doi: 10.1128/IAI.00806-12 PMC3486034 22927051

[97] Kamoshida G Kikuchi-Ueda T Nishida S Tansho-Nagakawa S Ubagai T Ono Y. Pathogenic bacterium *acinetobacter baumannii* inhibits the formation of neutrophil extracellular traps by suppressing neutrophil adhesion. Front Immunol (2018) 9:178. doi: 10.3389/fimmu.2018.00178 29467765 PMC5808340

[98] Chen Y Zhao C Guo H Zou W Zhang Z Wei D. Wip1 inhibits neutrophil extracellular traps to promote abscess formation in mice by directly dephosphorylating Coronin-1a. Cell Mol Immunol (2023) 20(8):941–54. doi: 10.1038/s41423-023-01057-2 PMC10387484 37386173

[99] Ali SR Fong JJ Carlin AF Busch TD Linden R Angata T. Siglec-5 and Siglec-14 are polymorphic paired receptors that modulate neutrophil and amnion signaling responses to group B Streptococcus. J Exp Med (2014) 211(6):1231–42. doi: 10.1084/jem.20131853 PMC4042635 24799499

[100] Lood C Blanco LP Purmalek MM Carmona-Rivera C De Ravin SS Smith CK. Neutrophil extracellular traps enriched in oxidized mitochondrial DNA are interferogenic and contribute to lupus-like disease. Nat Med (2016) 22(2):146–53. doi: 10.1038/nm.4027 PMC4742415 26779811

[101] Berends ET Horswill AR Haste NM Monestier M Nizet V von Köckritz-Blickwede M. Nuclease expression by *Staphylococcus aureus* facilitates escape from neutrophil extracellular traps. J innate immunity. (2010) 2(6):576–86. doi: 10.1159/000319909 PMC2982853 20829609

[102] Herzog S Dach F de Buhr N Niemann S Schlagowski J Chaves-Moreno D. High nuclease activity of long persisting *staphylococcus aureus* isolates within the airways of cystic fibrosis patients protects against NET-mediated killing. Front Immunol (2019) 10:2552. doi: 10.3389/fimmu.2019.02552 31772562 PMC6849659

[103] Eisenbeis J Saffarzadeh M Peisker H Jung P Thewes N Preissner KT. The *staphylococcus aureus* extracellular adherence protein eap is a DNA binding protein capable of blocking neutrophil extracellular trap formation. Front Cell infection Microbiol (2018) 8:235. doi: 10.3389/fcimb.2018.00235 PMC6047304 30038902

[104] Chavakis T Wiechmann K Preissner KT Herrmann M. *Staphylococcus aureus* interactions with the endothelium: the role of bacterial "secretable expanded repertoire adhesive molecules" (SERAM) in disturbing host defense systems. Thromb haemostasis. (2005) 94(2):278–85. doi: 10.1160/TH05-05-0306 16113816

[105] Thammavongsa V Missiakas DM Schneewind O. *Staphylococcus aureus* degrades neutrophil extracellular traps to promote immune cell death. Science. (2013) 342(6160):863–6. doi: 10.1126/science.1242255 PMC4026193 24233725

[106] Thammavongsa V Kern JW Missiakas DM Schneewind O. *Staphylococcus aureus* synthesizes adenosine to escape host immune responses. J Exp Med (2009) 206(11):2417–27. doi: 10.1084/jem.20090097 PMC2768845 19808256

[107] Kwiecinski JM Kratofil RM Parlet CP Surewaard BGJ Kubes P Horswill AR. *Staphylococcus aureus* uses the ArlRS and MgrA cascade to regulate immune evasion during skin infection. Cell Rep (2021) 36(4):109462. doi: 10.1016/j.celrep.2021.109462 34320352 PMC8450000

[108] Cordero M García-Fernández J. The induction of natural competence adapts staphylococcal metabolism to infection. Nat Commun (2022) 13(1):1525. doi: 10.1038/s41467-022-29206-7 35314690 PMC8938553

[109] Pietrocola G Nobile G Alfeo MJ Foster TJ Geoghegan JA De Filippis V. Fibronectin-binding protein B (FnBPB) from *Staphylococcus aureus* protects against the antimicrobial activity of histones. J Biol Chem (2019) 294(10):3588–602. doi: 10.1074/jbc.RA118.005707 PMC6416437 30622139

[110] Wilton M Halverson TWR Charron-Mazenod L Parkins MD Lewenza S. Secreted Phosphatase and Deoxyribonuclease Are Required by *Pseudomonas aeruginosa* To Defend against Neutrophil Extracellular Traps. Infection Immun (2018) 86(9):e00403-18. doi: 10.1128/IAI.00403-18 PMC6105888 29967090

[111] Tanner L Bhongir RKV Karlsson CAQ Le S Ljungberg JK Andersson P. Citrullination of extracellular histone H3.1 reduces antibacterial activity and exacerbates its proteolytic degradation. J cystic fibrosis Off J Eur Cystic Fibrosis Soc (2021) 20(2):346–55. doi: 10.1016/j.jcf.2020.07.010 32727663

[112] Moon AF Krahn JM Lu X Cuneo MJ Pedersen LC. Structural characterization of the virulence factor Sda1 nuclease from Streptococcus pyogenes. Nucleic Acids Res (2016) 44(8):3946–57. doi: 10.1093/nar/gkw143 PMC4856990 26969731

[113] Chang A Khemlani A Kang H Proft T. Functional analysis of *Streptococcus pyogenes* nuclease A (SpnA), a novel group A streptococcal virulence factor. Mol Microbiol (2011) 79(6):1629–42. doi: 10.1111/j.1365-2958.2011.07550.x 21231972

[114] Zhu L Kuang Z Wilson BA Lau GW. Competence-independent activity of pneumococcal EndA mediates degradation of extracellular dna and nets and is important for virulence. PloS One (2013) 8(7):e70363. doi: 10.1371/annotation/75f51a45-a2de-4893-94cf-0045056a4d7c 23936195 PMC3729463

[115] Jhelum H Sori H Sehgal D. A novel extracellular vesicle-associated endodeoxyribonuclease helps *Streptococcus pneumoniae* evade neutrophil extracellular traps and is required for full virulence. Sci Rep (2018) 8(1):7985. doi: 10.1038/s41598-018-25865-z 29789571 PMC5964101

[116] de Buhr N Neumann A Jerjomiceva N von Köckritz-Blickwede M Baums CG. *Streptococcus suis* DNase SsnA contributes to degradation of neutrophil extracellular traps (NETs) and evasion of NET-mediated antimicrobial activity. Microbiology. (2014) 160(2):385–95. doi: 10.1099/mic.0.072199-0 24222615

[117] de Buhr N Stehr M Neumann A Naim HY Valentin-Weigand P von Köckritz-Blickwede M. Identification of a novel DNase of *Streptococcus suis* (EndAsuis) important for neutrophil extracellular trap degradation during exponential growth. Microbiol . (2015) 161(4):838–50. doi: 10.1099/mic.0.000040 25667008

[118] Xie F Zan Y Zhang Y Zheng N Yan Q Zhang W. The cysteine protease ApdS from *Streptococcus suis* promotes evasion of innate immune defenses by cleaving the antimicrobial peptide cathelicidin LL-37. J Biol Chem (2019) 294(47):17962–77. doi: 10.1074/jbc.RA119.009441 PMC6879338 31619521

[119] Morita C Sumioka R Nakata M Okahashi N Wada S Yamashiro T. Cell wall-anchored nuclease of *Streptococcus sanguinis* contributes to escape from neutrophil extracellular trap-mediated bacteriocidal activity. PloS One (2014) 9(8):e103125. doi: 10.1371/journal.pone.0103125 25084357 PMC4118848

[120] Juneau RA Stevens JS Apicella MA Criss AK. A thermonuclease of *Neisseria gonorrhoeae* enhances bacterial escape from killing by neutrophil extracellular traps. J Infect diseases. (2015) 212(2):316–24. doi: 10.1093/infdis/jiv031 PMC4490236 25605868

[121] Möllerherm H Neumann A Schilcher K Blodkamp S Zeitouni NE Dersch P. *Yersinia enterocolitica*-mediated degradation of neutrophil extracellular traps (NETs). FEMS Microbiol Lett (2015) 362(23):fnv192. doi: 10.1093/femsle/fnv192 26459885

[122] Seper A Hosseinzadeh A Gorkiewicz G Lichtenegger S Roier S Leitner DR. *Vibrio cholerae* evades neutrophil extracellular traps by the activity of two extracellular nucleases. PloS pathogens. (2013) 9(9):e1003614. doi: 10.1371/journal.ppat.1003614 24039581 PMC3764145

[123] Scharrig E Carestia A Ferrer MF Cédola M Pretre G Drut R. Neutrophil extracellular traps are involved in the innate immune response to infection with leptospira. PloS Negl Trop Dis (2015) 9(7):e0003927. doi: 10.1371/journal.pntd.0003927 26161745 PMC4498591

[124] Kumar A Varma VP Sridhar K Abdullah M Vyas P Ashiq Thalappil M. Deciphering the role of *leptospira* surface protein ligA in modulating the host innate immune response. Front Immunol (2021) 12:807775. doi: 10.3389/fimmu.2021.807775 34975922 PMC8716722

[125] Kumar A Varma VP Faisal SM. Screening of surface-exposed lipoproteins of *leptospira* involved in modulation of host innate immune response. Front Microbiol (2022) 13:761670. doi: 10.3389/fmicb.2022.761670 35401498 PMC8988195

[126] Doke M Fukamachi H Morisaki H Arimoto T Kataoka H Kuwata H. Nucleases from *Prevotella intermedia* can degrade neutrophil extracellular traps. Mol Oral Microbiol (2017) 32(4):288–300. doi: 10.1111/omi.12171 27476978 PMC5516193

[127] Brogden G von Köckritz-Blickwede M Adamek M Reuner F Jung-Schroers V Naim HY. β-Glucan protects neutrophil extracellular traps against degradation by *Aeromonas hydrophila* in carp (Cyprinus carpio). Fish shellfish Immunol (2012) 33(4):1060–4. doi: 10.1016/j.fsi.2012.08.009 22959188

[128] Zang X Dang G Cai Z Shao M Tang Y Cao J. Extracellular DNase MAP3916c attacks the neutrophil extracellular traps and is needed for *Mycobacterium avium* subsp. paratuberculosis virulence. Veterinary Microbiol (2022) 273:109529. doi: 10.1016/j.vetmic.2022.109529 35944391

[129] Mitiku F Hartley CA Sansom FM Coombe JE Mansell PD Beggs DS. The major membrane nuclease MnuA degrades neutrophil extracellular traps induced by Mycoplasma bovis. Veterinary Microbiol (2018) 218:13–9. doi: 10.1016/j.vetmic.2018.03.002 29685215

[130] Zhang H Zhao G Guo Y Menghwar H Chen Y Chen H. *Mycoplasma bovis* MBOV_RS02825 encodes a secretory nuclease associated with cytotoxicity. Int J Mol Sci (2016) 17(5):628. doi: 10.3390/ijms17050628 27136546 PMC4881454

[131] Yamamoto T Kida Y Sakamoto Y Kuwano K. Mpn491, a secreted nuclease of *Mycoplasma pneumoniae*, plays a critical role in evading killing by neutrophil extracellular traps. Cell Microbiol (2017) 19(3):e12666. doi: 10.1111/cmi.12666 27603754

[132] Li P Zhang Y Li X Zhou W Li X Jiang F. *Mycoplasma hyopneumoniae* Mhp597 is a cytotoxicity, inflammation and immunosuppression associated nuclease. Veterinary Microbiol (2019) 235:53–62. doi: 10.1016/j.vetmic.2019.05.011 31282379

[133] Cacciotto C Dessì D Cubeddu T Cocco AR Pisano A Tore G. MHO_0730 as a surface-exposed calcium-dependent nuclease of *mycoplasma hominis* promoting neutrophil extracellular trap formation and escape. J Infect diseases. (2019) 220(12):1999–2008. doi: 10.1093/infdis/jiz406 31420650

[134] Stobernack T du Teil Espina M Mulder LM Palma Medina LM Piebenga DR Gabarrini G. A secreted bacterial peptidylarginine deiminase can neutralize human innate immune defenses. mBio. (2018) 9(5):10–128. doi: 10.1128/mBio.01704-18 PMC6212822 30377277

[135] Birnberg-Weiss F Castillo LA Pittaluga JR Martire-Greco D Gómez SA Landoni VI. Modulation of neutrophil extracellular traps release by Klebsiella pneumoniae. J leukocyte Biol (2021) 109(1):245–56. doi: 10.1002/JLB.4MA0620-099R 32640486

[136] Yang W. Nucleases: diversity of structure, function and mechanism. Q Rev biophysics. (2011) 44(1):1–93. doi: 10.1017/S0033583510000181 PMC6320257 20854710

[137] de Buhr N Bonilla MC Pfeiffer J Akhdar S Schwennen C Kahl BC. Degraded neutrophil extracellular traps promote the growth of Actinobacillus pleuropneumoniae. Cell Death Dis (2019) 10(9):657. doi: 10.1038/s41419-019-1895-4 31506432 PMC6736959

[138] Cacciotto C Alberti A. Eating the enemy: mycoplasma strategies to evade neutrophil extracellular traps (NETs) promoting bacterial nucleotides uptake and inflammatory damage. Int J Mol Sci (2022) 23(23):15030. doi: 10.3390/ijms232315030 36499356 PMC9740415

[139] Benedetti F Curreli S Zella D. Mycoplasmas-host interaction: Mechanisms of inflammation and association with cellular transformation. Microorganisms. (2020) 8(9):1351. doi: 10.3390/microorganisms8091351 32899663 PMC7565387

[140] Masukagami Y De Souza DP Dayalan S Bowen C O'Callaghan S Kouremenos K. Comparative Metabolomics of *Mycoplasma bovis* and *Mycoplasma gallisepticum* Reveals Fundamental Differences in Active Metabolic Pathways and Suggests Novel Gene Annotations. mSystems. (2017) 2(5):e00055-17. doi: 10.1128/mSystems.00055-17 29034329 PMC5634790

[141] Yiwen C Yueyue W Lianmei Q Cuiming Z Xiaoxing Y. Infection strategies of mycoplasmas: Unraveling the panoply of virulence factors. Virulence. (2021) 12(1):788–817. doi: 10.1080/21505594.2021.1889813 33704021 PMC7954426

[142] Yueyue W Feichen X Yixuan X Lu L Yiwen C Xiaoxing Y. Pathogenicity and virulence of *Mycoplasma genitalium*: Unraveling Ariadne's Thread. Virulence. (2022) 13(1):1161–83. doi: 10.1080/21505594.2022.2095741 PMC9262362 35791283

[143] Yacoub E Ben Abdelmoumen Mardassi B. Mm19, a *mycoplasma meleagridis* major surface nuclease that is related to the RE_AlwI superfamily of endonucleases. PloS One (2016) 11(3):e0152171. doi: 10.1371/journal.pone.0152171 27010566 PMC4807054

[144] Li L Krishnan M Baseman JB Kannan TR. Molecular cloning, expression, and characterization of a Ca^2+^-dependent, membrane-associated nuclease of Mycoplasma genitalium. J bacteriology (2010) 192(19):4876–84. doi: 10.1128/JB.00401-10 PMC2944508 20639320

[145] Cacciotto C Addis MF Coradduzza E Carcangiu L Nuvoli AM Tore G. *Mycoplasma agalactiae* MAG_5040 is a Mg2+-dependent, sugar-nonspecific SNase recognised by the host humoral response during natural infection. PloS One (2013) 8(2):e57775. doi: 10.1371/journal.pone.0057775 23469065 PMC3585158

[146] Xu J Teng D Jiang F Zhang Y El-Ashram SA Wang H. *Mycoplasma gallisepticum* MGA_0676 is a membrane-associated cytotoxic nuclease with a staphylococcal nuclease region essential for nuclear translocation and apoptosis induction in chicken cells. Appl Microbiol Biotechnol (2015) 99(4):1859–71. doi: 10.1007/s00253-014-6185-6 25363559

[147] Tilvawala R Thompson PR. Peptidyl arginine deiminases: detection and functional analysis of protein citrullination. Curr Opin Struct Biol (2019) 59:205–15. doi: 10.1016/j.sbi.2019.01.024 PMC6717693 30833201

[148] Li P Li M Lindberg MR Kennett MJ Xiong N Wang Y. PAD4 is essential for antibacterial innate immunity mediated by neutrophil extracellular traps. J Exp Med (2010) 207(9):1853–62. doi: 10.1084/jem.20100239 PMC2931169 20733033

[149] Pachón-Ibáñez ME Smani Y Pachón J Sánchez-Céspedes J. Perspectives for clinical use of engineered human host defense antimicrobial peptides. FEMS Microbiol Rev (2017) 41(3):323–42. doi: 10.1093/femsre/fux012 PMC5435762 28521337

[150] Wartha F Beiter K Albiger B Fernebro J Zychlinsky A Normark S. Capsule and D-alanylated lipoteichoic acids protect *Streptococcus pneumoniae* against neutrophil extracellular traps. Cell Microbiol (2007) 9(5):1162–71. doi: 10.1111/j.1462-5822.2006.00857.x 17217430

[151] Reid SD Hong W Dew KE Winn DR Pang B Watt J. *Streptococcus pneumoniae* forms surface-attached communities in the middle ear of experimentally infected chinchillas. J Infect diseases. (2009) 199(6):786–94. doi: 10.1086/597042 19434911

[152] Muñoz VL Porsch EA St Geme JW 3rd. *Kingella kingae* surface polysaccharides promote resistance to neutrophil phagocytosis and killing. mBio. (2019) 10(3):e00631–19. doi: 10.1128/mBio.00631-19 PMC6593399 31239373

[153] LaRock CN Döhrmann S Todd J Corriden R Olson J Johannssen T. Group A streptococcal M1 protein sequesters cathelicidin to evade innate immune killing. Cell Host Microbe (2015) 18(4):471–7. doi: 10.1016/j.chom.2015.09.004 PMC4636435 26468750

[154] Chen YH Li SH Yang YC Hsu SH Nizet V. T4 pili promote colonization and immune evasion phenotypes of nonencapsulated M4 streptococcus pyogenes. mBio (2020) 11(4):e01580–20. doi: 10.1128/mBio.01580-20 PMC7374061 32694142

[155] Cole JN Pence MA von Köckritz-Blickwede M Hollands A Gallo RL Walker MJ. M protein and hyaluronic acid capsule are essential for in *vivo* selection of covRS mutations characteristic of invasive serotype M1T1 group A Streptococcus. mBio (2010) 1(4):e00191–10. doi: 10.1128/mBio.00191-10 PMC2934611 20827373

[156] Döhrmann S Anik S Olson J Anderson EL Etesami N No H. Role for streptococcal collagen-like protein 1 in M1T1 group A *Streptococcus* resistance to neutrophil extracellular traps. Infection immunity. (2014) 82(10):4011–20. doi: 10.1128/IAI.01921-14 PMC4187857 25024366

[157] Juneau RA Pang B Weimer KE Armbruster CE Swords WE. Nontypeable *Haemophilus influenzae* initiates formation of neutrophil extracellular traps. Infection immunity. (2011) 79(1):431–8. doi: 10.1128/IAI.00660-10 PMC3019868 20956567

[158] Juneau RA Pang B Armbruster CE Murrah KA Perez AC Swords WE. Peroxiredoxin-glutaredoxin and catalase promote resistance of nontypeable *Haemophilus influenzae* 86-028NP to oxidants and survival within neutrophil extracellular traps. Infection immunity. (2015) 83(1):239–46. doi: 10.1128/IAI.02390-14 PMC4288874 25348637

[159] Shan Q Dwyer M Rahman S Gadjeva M. Distinct susceptibilities of corneal *Pseudomonas aeruginosa* clinical isolates to neutrophil extracellular trap-mediated immunity. Infection immunity. (2014) 82(10):4135–43. doi: 10.1128/IAI.02169-14 PMC4187885 25047845

[160] Ma F Yi L Yu N Wang G Ma Z Lin H. *Streptococcus suis* serotype 2 biofilms inhibit the formation of neutrophil extracellular traps. Front Cell infection Microbiol (2017) 7:86. doi: 10.3389/fcimb.2017.00086 PMC5357632 28373968

[161] Paton JC Trappetti C. *Streptococcus pneumoniae* capsular polysaccharide. Microbiol spectrum. (2019) 7(2):10–128. doi: 10.1128/microbiolspec.GPP3-0019-2018 PMC11590643 30977464

[162] Moorthy AN Rai P Jiao H Wang S Tan KB Qin L. Capsules of virulent pneumococcal serotypes enhance formation of neutrophil extracellular traps during in *vivo* pathogenesis of pneumonia. Oncotarget. (2016) 7(15):19327–40. doi: 10.18632/oncotarget.8451 PMC4991386 27034012

[163] Rather MA Gupta K Mandal M. Microbial biofilm: formation, architecture, antibiotic resistance, and control strategies. Braz J Microbiol (2021) 52(4):1701–18. doi: 10.1007/s42770-021-00624-x PMC8578483 34558029

[164] Chiang WC Nilsson M Jensen P Høiby N Nielsen TE Givskov M. Extracellular DNA shields against aminoglycosides in *Pseudomonas aeruginosa* biofilms. Antimicrobial Agents chemotherapy. (2013) 57(5):2352–61. doi: 10.1128/AAC.00001-13 PMC3632962 23478967

[165] Tseng BS Zhang W Harrison JJ Quach TP Song JL Penterman J. The extracellular matrix protects *Pseudomonas aeruginosa* biofilms by limiting the penetration of tobramycin. Environ Microbiol (2013) 15(10):2865–78. doi: 10.1111/1462-2920.12155 PMC4045617 23751003

[166] Mulcahy H Charron-Mazenod L Lewenza S. Extracellular DNA chelates cations and induces antibiotic resistance in *Pseudomonas aeruginosa* biofilms. PloS pathogens. (2008) 4(11):e1000213. doi: 10.1371/journal.ppat.1000213 19023416 PMC2581603

[167] Walker TS Tomlin KL Worthen GS Poch KR Lieber JG Saavedra MT. Enhanced *Pseudomonas aeruginosa* biofilm development mediated by human neutrophils. Infection immunity. (2005) 73(6):3693–701. doi: 10.1128/IAI.73.6.3693-3701.2005 PMC1111839 15908399

[168] Swords WE Moore ML Godzicki L Bukofzer G Mitten MJ VonCannon J. Sialylation of lipooligosaccharides promotes biofilm formation by nontypeable Haemophilus influenzae. Infection Immun (2004) 72(1):106–13. doi: 10.1128/IAI.72.1.106-113.2004 PMC343998 14688087

[169] Hong W Pang B West-Barnette S Swords WE. Phosphorylcholine expression by nontypeable *Haemophilus influenzae* correlates with maturation of biofilm communities in *vitro* and in vivo. J bacteriology (2007) 189(22):8300–7. doi: 10.1128/JB.00532-07 PMC2168690 17573475

[170] Greiner LL Watanabe H Phillips NJ Shao J Morgan A Zaleski A. Nontypeable Haemophilus influenzae strain 2019 produces a biofilm containing N-acetylneuraminic acid that may mimic sialylated O-linked glycans. Infection immunity. (2004) 72(7):4249–60. doi: 10.1128/IAI.72.7.4249-4260.2004 PMC427468 15213170

[171] Jurcisek JA Bookwalter JE Baker BD Fernandez S Novotny LA Munson RS Jr.. The PilA protein of non-typeable *Haemophilus influenzae* plays a role in biofilm formation, adherence to epithelial cells and colonization of the mammalian upper respiratory tract. Mol Microbiol (2007) 65(5):1288–99. doi: 10.1111/j.1365-2958.2007.05864.x 17645732

[172] Jurcisek JA Bakaletz LO. Biofilms formed by nontypeable *Haemophilus influenzae in vivo* contain both double-stranded DNA and type IV pilin protein. J bacteriology. (2007) 189(10):3868–75. doi: 10.1128/JB.01935-06 PMC1913342 17322318

[173] Bhattacharya M Berends ETM Zheng X Hill PJ Chan R Torres VJ. Leukocidins and the nuclease nuc prevent neutrophil-mediated killing of *staphylococcus aureus* biofilms. Infection immunity. (2020) 88(10):e00372–20. doi: 10.1128/IAI.00372-20 PMC7504955 32719153

[174] Lieberman LA. Outer membrane vesicles: A bacterial-derived vaccination system. Front Microbiol (2022) 13:1029146. doi: 10.3389/fmicb.2022.1029146 36620013 PMC9811673

[175] Cole JN Nizet V. Bacterial evasion of host antimicrobial peptide defenses. Microbiol Spectr. (2016) 4(1):10.1128/microbiolspec.VMBF-0006-2015. doi: 10.1128/microbiolspec.VMBF-0006-2015 PMC4804471 26999396

[176] Yang H Wang H Levine YA Gunasekaran MK Wang Y Addorisio M. Identification of CD163 as an antiinflammatory receptor for HMGB1-haptoglobin complexes. JCI Insight (2018) 3(24):e126617. doi: 10.1172/jci.insight.126617 30568039 PMC6338310

[177] Arredouani MS Kasran A Vanoirbeek JA Berger FG Baumann H Ceuppens JL. Haptoglobin dampens endotoxin-induced inflammatory effects both in *vitro* and in vivo. Immunology (2005) 114(2):263–71. doi: 10.1111/j.1365-2567.2004.02071.x PMC1782073 15667571

[178] Murdoch CC Skaar EP. Nutritional immunity: the battle for nutrient metals at the host-pathogen interface. Nat Rev Microbiol (2022) 20(11):657–70. doi: 10.1038/s41579-022-00745-6 PMC9153222 35641670

[179] Stork M Bos MP Jongerius I de Kok N Schilders I Weynants VE. An outer membrane receptor of *Neisseria meningitidis* involved in zinc acquisition with vaccine potential. PloS pathogens. (2010) 6(7):e1000969. doi: 10.1371/journal.ppat.1000969 20617164 PMC2895646

[180] Parker H Albrett AM Kettle AJ Winterbourn CC. Myeloperoxidase associated with neutrophil extracellular traps is active and mediates bacterial killing in the presence of hydrogen peroxide. J leukocyte Biol (2012) 91(3):369–76. doi: 10.1189/jlb.0711387 22131345

