# Supplementary material for: Neutrophil extracellular traps in bacterial infections and evasion strategies
Source: Front Immunol. 2024 Feb 16;15:1357967. doi: 10.3389/fimmu.2024.1357967 (PMC10906519; doi:10.3389/fimmu.2024.1357967)
Supplement: Supplementary file 1 [file DataSheet_1.docx]

**Table S1 The key protein components of NETs**

| **Cellular localization** | | **Protein name** | **References** |
| --- | --- | --- | --- |
| Nucleus | | Histones (H1, H2A, H2B, H3, H4) | (7,52,53) |
| Granules | Primary granules | Neutrophil elastase | (7,52,53) |
|  |  | Myeloperoxidase | (7,52,53) |
|  |  | Cathepsin G | (7,52,53) |
|  |  | Defensins | (7,52,53) |
|  | Secondary granules | Lactoferrin | (7,52,53) |
|  |  | Cathelicidin | (52,53) |
|  |  | Lysozyme C | (52,53) |
|  | Tertiary granules | Gelatinase | (7,53) |
| Cytoplasm | | Calprotectin protein complex | (52,53) |
|  |  | proteinase-3 | (52,53) |
